# Mediterranean-DASH Intervention for Neurodegenerative Delay (MIND) Trial: Genetic Resource for Precision Nutrition

**DOI:** 10.3390/nu17152548

**Published:** 2025-08-04

**Authors:** Yuxi Liu, Hailie Fowler, Dong D. Wang, Lisa L. Barnes, Marilyn C. Cornelis

**Affiliations:** 1 Channing Division of Network Medicine, Department of Medicine, Brigham and Women’s Hospital and Harvard Medical School, Boston, MA 02115, USA; yuxiliu@mail.harvard.edu (Y.L.); dow471@mail.harvard.edu (D.D.W.); 2 Department of Epidemiology, Harvard T.H. Chan School of Public Health, Boston, MA 02115, USA; 3 Broad Institute of MIT and Harvard, Cambridge, MA 02142, USA; 4 NUSeq Core Facility, Northwestern University Feinberg School of Medicine, Chicago, IL 60611, USA; hailie.fowler@northwestern.edu; 5 Department of Nutrition, Harvard T.H. Chan School of Public Health, Boston, MA 02115, USA; 6 Department of Neurological Sciences, Rush University Medical Center, Chicago, IL 60612, USA; lisa_l_barnes@rush.edu; 7 Rush Alzheimer’s Disease Center, Chicago, IL 60612, USA; 8 Department of Preventive Medicine, Northwestern University Feinberg School of Medicine, Chicago, IL 60611, USA

**Keywords:** diet pattern, dementia, genotype, quality control, clinical trial

## Abstract

**Background:** The Mediterranean-DASH Intervention for Neurodegenerative Delay (MIND) was a 3-year, multicenter, randomized controlled trial to test the effects of the MIND diet on cognitive decline in 604 individuals at risk for Alzheimer’s dementia. Here, we describe the genotyping, imputation, and quality control (QC) procedures for the genetic data of trial participants. **Methods:** DNA was extracted from either whole blood or serum, and genotyping was performed using the Infinium Global Diversity Array. Established sample and SNP QC procedures were applied to the genotyping data, followed by imputation using the 1000 Genomes Phase 3 v5 reference panel. **Results:** Significant study-site, specimen type, and batch effects were observed. A total of 494 individuals of inferred European ancestry and 58 individuals of inferred African ancestry were included in the final imputed dataset. Evaluation of the imputed *APOE* genotype against gold-standard sequencing data showed high concordance (98.2%). We replicated several known genetic associations identified from previous genome-wide association studies, including SNPs previously linked to adiponectin (rs16861209, *p* = 1.5 × 10^−5^), alpha-linolenic acid (rs174547, *p* = 1.3 × 10^−7^), and alpha-tocopherol (rs964184, *p* = 0.003). **Conclusions:** This dataset represents the first genetic resource derived from a dietary intervention trial focused on cognitive outcomes. It enables investigation of genetic contributions to variability in cognitive response to the MIND diet and supports integrative analyses with other omics data types to elucidate the biological mechanisms underlying cognitive decline. These efforts may ultimately inform precision nutrition strategies to promote cognitive health.

## 1. Introduction

The prevalence of dementia, particularly due to Alzheimer’s disease (AD), is expected to increase due to the progressive aging of the world population [1]. Age-associated cognitive decline varies in extent among individuals, and this variation likely arises from a complex interplay of genetic and environmental factors. The Mediterranean-DASH Intervention for Neurodegenerative Delay (MIND) diet [2] is a hybrid of the Mediterranean diet (MeDi) and Dietary Approaches to Stop Hypertension (DASH) diet, but with selected modifications based on the most compelling evidence in the diet-dementia field [3]. MIND emphasizes consuming green leafy vegetables, berries, nuts, beans, whole grains, seafood, poultry, olive oil, wine, and limited intake of animal and high-saturated-fat foods. Population studies suggest adherence to MIND may slow the rate of cognitive decline and decrease the risk of AD [2,4].

Results of the first randomized controlled trial (RCT) of a calorie-restricted MIND diet on cognitive change were reported in 2023 [5]. No significant differences in cognitive change after 3 years were observed compared to a calorie-restricted control diet. Both arms lost weight and presented with significant improvements in cognition, thus emphasizing a key role of weight management in brain health. Between-person variation in metabolism of constituents characteristic of MIND may enhance or limit their efficacy and ultimately impact response to this diet. Knowledge of genetic factors contributing to this variation is particularly useful, as such factors can (i) serve as tools to understand mechanisms underlying the relationship between MIND and cognitive health, and (ii) be used to identify subgroups of individuals who especially benefit when adhering to this diet.

The current study describes the genetic data complementing the trial and specifically the challenges faced in acquiring the data due, in part, to the COVID-19 pandemic.

## 2. Materials and Methods

### 2.1. The MIND Trial

The MIND trial was a 3-year, two-site, investigator-blinded phase III RCT of the effect of the MIND diet with mild caloric restriction (goal 250 kcal less/day) for weight loss compared to the usual diet with the same caloric restriction for weight loss (control diet) on cognitive decline. The trial design and results have been published previously [5,6]. The trial had two clinical sites, Chicago (Rush University) and Boston (Harvard University), and recruited older individuals (65+) living within these areas who were cognitively unimpaired, overweight, and had a family history of dementia and a suboptimal diet (MIND score ≤ 8 out of 14). Recruitment efforts included mass mailings and advertisements on public transport, worksites, the radio, and newspapers. Interested individuals were pre-screened by telephone, followed by an in-person screening for informed consent and remaining eligibility criteria. Those determined eligible completed a 3- to 4-week run-in to assess their likelihood of complying with trial interventions. Participants who successfully completed the run-in were invited back to complete a 2-day baseline visit. Measures of height, weight, and a fasting blood sample were collected, cognition tests were performed, and questionnaires were completed. Participants from one site wore a physical activity sensor for 7 consecutive days. All participants were randomly assigned by the Data Coordinating Center in a 1:1 ratio to the MIND-diet group or the control-diet group; stratified by trial site, sex, and 4 age categories (65–69, 70–74, 75–80, and 81–84 years). Twenty-four-hour dietary recalls and blood and urine biomarkers of diet were measured at baseline and over the course of follow-up.

The trial followed the ethical standards of the 1964 Declaration of Helsinki and its later amendments and was approved by the institutional review boards at Rush University Medical Center, Harvard School of Public Health, and Brigham and Women’s Hospital. All the participants provided written informed consent. Participants were enrolled from January 2017 to April 2018; data collection ended June 2021. A total of 604 participants were enrolled and randomized. All phenotype data used for the current study were collected at baseline. Blood samples at various time points were considered for DNA extraction as detailed below. We remained blinded to participant randomization throughout the generation of the genetic data.

### 2.2. Coronavirus Disease 2019 (COVID-19)

During the pandemic, in-person research was suspended in March 2020 and resumed in mid July 2020. The economical fall resulting from COVID-19 also forced the closure of the laboratory initially charged with performing *APOE* genotyping (see Box 1 for glossary of terms). *APOE* genotyping of baseline whole blood samples was completed for 231 trial participants, but residual DNA was not saved upon lab closure. The second laboratory completed the *APOE* genotyping for the remaining 373 trial participants and residual DNA saved. The latter was thus available for genotyping. Whole bloods were not available for participants assayed at the first lab, so we proceeded with DNA extractions from serum and testing their performance for genotyping. All participants were enrolled and baseline measures taken prior to the pandemic, thus COVID-19 would not have impacted the genetic data collected or baseline measures examined in the current study.

Box 1 **Glossary of Terms.**

  ***APOE*:** A gene located on chromosome 19 that encodes apolipoprotein E, which is involved in lipid transport and metabolism. Genetic variation in *APOE*, particularly the ε4 allele, is the strongest genetic risk factor for Alzheimer’s disease and cognitive decline.

  **Allele dosage:** The number of copies of a specific allele that an individual carries at a given genetic locus, typically ranging from 0 to 2 for biallelic variants. In imputed datasets, allele dosages are often non-integer and reflect the expected number of copies based on genotype probabilities.

  **Autosomal biallelic SNPs:** Single nucleotide polymorphisms (SNPs) located on the autosomes (chromosomes 1–22) that have two alleles in the population. These are the most commonly studied type of genetic variant in genome-wide association studies (GWAS).

  **Call rate:** The proportion of genetic variants or samples that are successfully genotyped. Low call rates may indicate poor data quality and are commonly used in quality control.

  **Genotype:** The set of alleles an individual carries at a specific genetic locus. Genotypes are typically classified as homozygous reference (two copies of the reference allele), heterozygous (one reference and one alternate allele), or homozygous alternate (two copies of the alternate allele).

  **Genome-wide association studies (GWAS):** Studies that examine genetic variants across the entire genome to identify those associated with specific traits or diseases.

  **GRCh37/GRCh38 reference genome:** Versions of the human reference genome assembly used as standardized coordinate systems for aligning sequencing data and reporting genetic variant positions. GRCh37 (also known as hg19) and GRCh38 (hg38) are the most commonly used builds.

  **Hardy–Weinberg equilibrium (HWE):** Under assumptions of random mating, no selection, mutation, migration, or genetic drift, allele and genotype frequencies in a population will remain constant across generations. Deviations from HWE can suggest genotyping error, population stratification, or other violations of these assumptions.

  **Identity-by-descent (IBD):** A measure of genetic sharing indicating whether individuals inherited a segment of DNA from a common ancestor. IBD estimation is used to identify related individuals and assess familial relationships.

  **Imputation:** A statistical method used to infer untyped genotypes by leveraging known linkage disequilibrium patterns from a reference panel. Imputation increases genomic coverage and improves harmonization across datasets genotyped on different platforms.

  **Imputation quality:** A measure of confidence in imputed genotypes, commonly quantified by the INFO score (R^2^), which ranges from 0 to 1. Higher values indicate greater reliability of the imputed genotype, with values close to 1 suggesting high concordance with true genotypes.

  **Inbreeding coefficient:** A measure of the genetic similarity between an individual’s two alleles at a locus, reflecting the probability of inheritance from a common ancestor. High inbreeding coefficients may indicate consanguinity or population bottlenecks.

  **Kinship:** A metric quantifying the genetic relatedness between pairs of individuals. Kinship coefficients are used to control for familial structure in genetic analyses.

  **Linkage disequilibrium (LD):** The occurrence of alleles at two or more genetic loci together more or less frequently in a population than expected from their individual allele frequencies, typically because the loci are physically close on a chromosome and influenced by recombination, selection, genetic drift, and population structure.

  **Minor allele frequency (MAF):** The frequency of the less common allele at a genetic locus in a given population.

  **Population stratification:** Systematic allele frequency differences between populations due to underlying ancestry or genetic structure rather than association with the phenotype of interest.

  **Runs of homozygosity (ROH):** Long stretches of consecutive homozygous genotypes, reflecting inheritance of identical haplotypes from both parents. ROH indicate autozygosity and may result from inbreeding, population bottlenecks, or genetic drift.

  **Variant:** A genomic position that differs from the reference sequence, including SNPs, insertions, deletions, and structural changes. Variants are often classified by MAF: common (MAF ≥ 5%), low frequency (1% ≤ MAF < 5%), and rare (MAF < 1%).

### 2.3. DNA Extraction and Genotyping

Extraction of genomic DNA from whole blood samples was performed using the Wizard Genomic DNA purification kit (Promega Inc., Madison, WI, USA) following manufacturer guidelines [7]. The majority of serum samples for DNA extractions were obtained from blood collections at baseline (57%) and month 3 (37%); the remaining (5%) from later timepoints. Extraction of genomic DNA from serum samples was performed using the QIAamp DSP Virus Spin purification kit (Qiagen, Hilden, Germany) following standard manufacturer guidelines. Four participants had DNA extracted from both serum and blood samples that was subsequently used for testing genotyping performance. Genotyping of trial samples and male HapMap controls (5 CEPH/NA12801, 2 AASW/NA19711) was performed using Infinium Global Diversity Array 8 v1.0 (GDA, Illumina, San Diego, CA, USA, total assays = 1,904,599). For samples with sufficient DNA yield, the recommended input of 200 ng DNA was used; otherwise, the full volume of available DNA was used. Other than this varied input amount, the manufacturer’s standard instructions were followed for processing the arrays. Raw data were evaluated using GenomeStudio Controls Dashboard to assess if processing was successful. Genetic data were aligned to the GRCh38 reference genome. Additional QC described below was performed using PLINK1.9 and PLINK2.0 [8,9] and the R software v4.4.1 [10]. Prior to in-depth QC, we excluded all samples with call rates < 90% and all SNPs with minor allele frequencies (MAF) < 0.01.

### 2.4. Sample QC1: Sex-Check, Relatedness, and Effects of Array Batch, Specimen, and Study-Site

For sample QC, we used SNPs with call rates > 95%, MAF > 0.05, and Hardy–Weinberg equilibrium (HWE) *p* > 1 × 10^−6^. We identified mismatches between genetically inferred sex and self-reported sex using the PLINK command --check-sex. We examined relatedness (kinship, initial round) among our trial samples by generating a relationship matrix using the PLINK command --make-rel. Estimated kinship coefficient range > 0.354, [0.177, 0.354], [0.0884, 0.177] and [0.0442, 0.0884] corresponds to duplicate/MZ twin, 1st-degree, 2nd-degree, and 3rd-degree relationships, respectively. Effects of array batch, DNA specimen (blood, serum), and study-site (Rush, Harvard) were assessed by comparing the sample call rates, computed in PLINK using --missing, across batch, specimen, and study-site, respectively. Specimen performance was also assessed by estimating identity-by-descent (IBD) and genotype concordance (Section 2.6) among (expected) duplicate blood–blood pairs (HapMap only), serum–serum pairs (trial only), and blood–serum pairs (trial only). IBD was estimated using --keep [paired samples] --genome full functions in PLINK to calculate PI_HAT, the proportion of the genome shared identical-by-descent. Pairs with PI_HAT > 0.98 were considered genetically identical [11].

### 2.5. Ancestry

The inclusion of samples with diverse ancestry can confound QC metrics, largely due to ancestry differences in allele frequency distributions. Thus, prior to SNP-based QC, we applied principal component analysis (PCA) to genetically infer ancestry of our samples and to compare this designation with self-reported race. For this preliminary PCA, we used unrelated samples (i.e., excluded 2nd-degree relatives and closer, and expected duplicates) and a subset of independent autosomal biallelic SNPs with call rates > 95%, MAF > 0.05, and HWE *p* > 1 × 10^−6^ by applying PLINK’s filtering and clumping procedures (--snps-only, --autosome, --geno 0.05, --maf 0.05, --hwe 1 × 10^−6^, --indep-pairwise (50, 5 SNPs, r2 0.1)) [12]. PCA was performed in PLINK2 using --pca. We used the results of this analysis to perform SNP-QC and a second pass kinship analysis by ancestry.

### 2.6. SNP QC1: Genotype Concordance

Genotyping reproducibility was estimated by evaluating genotype concordance between duplicate pairs. We used only duplicate pairs with call rates > 95% and similar ancestry: specifically, samples of European ancestry (largest group) identified by PCA. Genotype data for each duplicate pair were exported using PLINK (--recode A), and pairwise genotype concordance was assessed directly in R. Analyses were restricted to autosomal biallelic SNPs. SNPs were further excluded from comparison if both samples were heterozygotes (i.e., double-heterozygous), as these genotypes are uninformative for detecting mismatches. Concordance rates were calculated as the proportion of concordant genotypes among all comparable SNPs. SNPs that were discordant were flagged. All other SNP-level QC procedures were performed after sample-level filtering (Section 2.8).

### 2.7. Sample QC2: Runs of Homozygosity (ROH)

Extreme heterozygosity and/or low call rate can be indicators of poor sample quality. Some samples can have naturally extreme heterozygosity, even after accounting for population structure (i.e., adjusting for PCs within a genetically inferred ancestry group). Specifically, individuals with mixed ethnicity tend to have higher heterozygosity (which is not captured by the PCs), and individuals whose parents are closely related tend to have lower heterozygosity. To distinguish between poor-quality samples and samples with naturally low heterozygosity, we looked for long ROH using PLINK: “--homozyg --homozyg-window-snp 50 --homozyg-snp 100 --homozyg-kb 1000 --homozyg-density 50 --homozyg-gap 1000” within each ancestry group. Heterozygosity was calculated using PLINK: “--het” per ancestry group. Samples with unusually short total ROH relative to others with similar heterozygosity were identified as outliers based on residuals from a LOESS regression of total ROH (in kilobases) on inbreeding coefficient (F). The LOESS model was fit separately within each ancestry group using a smoothing span of 0.5. Outliers were defined as samples with residuals below the 1st percentile of the residual distribution within each ancestry group.

### 2.8. SNP QC2: Final Variant Filtering

Having identified a set of high-quality, unrelated samples within each ancestry group with call rates > 90%, we performed a second round of SNP QC to retain autosomal and chromosome X variants with call rates > 95%, MAF > 0.01, and HWE *p* > 1 × 10^−6^ using PLINK (--geno 0.05, --maf 0.01, --hwe 1 × 10^−6^).

### 2.9. Population Substructure

We conducted a second round of PCA per ancestry with a second pruned set of autosomal biallelic SNPs with call rates > 98% and MAF > 0.05 using PLINK (--snps-only, --autosome, --geno 0.02, --maf 0.05, --hwe 1 × 10^−6^, --indep-pairwise (50, 5 SNPs, r2 0.1). PCA was performed in PLINK2 using --pca.

### 2.10. SNP Imputation

To prepare for imputation, variant coordinates were lifted over to the GRCh37 reference build using BCFtools + liftover [13]. Genotype imputation was conducted separately for each ancestry group using the Michigan Imputation Server 2 [14], using both the 1000 Genomes Project Phase 3 v5 (1000G) [15] and the Haplotype Reference Consortium (HRC) r1.1 2016 [16] reference panels (GRCh37/hg19). Post-imputation quality for each reference panel was assessed for autosomal variants (chromosomes 1–22) within each ancestry group. Variants were stratified by MAF bins (rare variants: <1%, low-frequency variants: 1–5%, common variants: ≥5%) and imputation INFO scores (R^2^ ≥ 0.3, 0.5, 0.8, 0.9). Imputed dosages were converted to PLINK2 format (--make-pgen) for downstream analyses.

### 2.11. Validity of Genetic Data

*APOE* genotype of trial participants was initially determined by sequencing rs429358 and rs7412 at exon 4 of *APOE*. We derived *APOE* genotype using imputed SNPs and assessed concordance with sequenced *APOE* genotype. We sought replication in the MIND trial sample of the strongest previously published loci for serum folate (2 SNPs tested), serum B12 (9 SNPs), serum alpha- (3 SNPs) and gamma- (2 SNPs) tocopherol, serum lycopene (1 SNP), serum alpha- (3 SNPs) and beta- (1 SNP) carotene, serum retinol (4 SNPs), plasma alpha-linolenic acid (ALA, 2 SNPs), plasma eicosapentaenoic acid (EPA, 4 SNPs), plasma docosahexaenoic acid (DHA, 17 SNPs), serum interleukin-6 (IL6, 3 SNPs), serum adiponectin (5 SNPs), body mass index (BMI, 31 SNPs) and self-reported macronutrient (12 SNPs) and coffee/tea/caffeine intake (2 SNPs). Effect size units were not compared since prior studies applied a variety of transformations. For MIND traits, data values were excluded if they failed assay technical QC (folate, adiponectin, B12) or were truncated to ±4 standard deviation (SD) of mean (BMI, IL6, carotenes, tocopherols, fatty acids) to ensure a normal distribution. Successful replication was defined as *p* < 0.05 and directional consistency. Trial assays, diet collection, and nutrient derivation have been described previously [5,6].

## 3. Results

### 3.1. DNA Extraction and Genotyping

DNA concentrations from serum ranged from 0.12 to 4.14 ng/μL. As expected, these were much lower than those for whole-blood extractions (4.4 to 277.8 ng/μL) (see Section 3.2 for specimen QC). We generated genetic data passing lab-level technical QC for 602 MIND trial samples (10 duplicate pairs) and seven male HapMap controls (five CEPH/NA12801, two AASW/NA19711). Thus, 12 of the total 604 trial participants had no genetic data, largely due to missing or low input amounts of DNA. Prior to in-depth QC, we applied two filters: sample call rates < 90% (19 samples excluded, all serum) and SNPs with MAF < 0.01 (n = 889,004), leaving 573 trial participants (10 genotyped twice) with genotype data for 1,015,595 SNPs. There were 79 array batches of six to eight samples.

### 3.2. Sample QC1: Sex-Check, Relatedness, and Effects of Array Batch, Specimen, and Study Site

**Figure 1:**
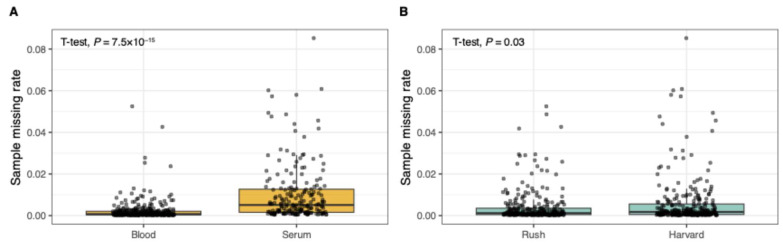
Sample missing rate by specimen type and study site. (**A**) By specimen type (blood, serum). (**B**) By study site (Rush University, Harvard University).

A subset of 630,959 SNPs with call rates > 95%, MAF > 0.05, and HWE *p* > 1 × 10^−6^ was used for these sample QC checks. We identified and dropped two samples with mismatches between genetically inferred sex and self-reported sex. Possible explanations for mismatches include clerical error (sample swap, coding entry), true mismatch, or sex chromosome aneuploidy (not assessed here). Trial staff were not aware of participants’ relatedness. We identified three pairs of first-degree relatives (siblings) that were highly plausible (date of enrollment and study site) and updated the pedigree accordingly. SNP call rates for serum ranged from 97.1% to 98.9% and for blood, 99.6% to 99.9%. Four participants had DNA extracted from both serum and blood samples; genotype concordance was > 99.2% for these blood–serum pairs. Expected duplicate blood–blood pairs, serum–serum pairs, and blood–serum pairs showed high genetic relatedness, with PI_HAT > 0.99 (IBD estimates from PLINK), further indicating consistent genotyping quality across specimen types. Nevertheless, sample missing rate by specimen type was significantly different (Figure 1A). We also observed significant differences in sample missing rate by study site (Figure 1B) even when accounting for the larger number of serum samples from Harvard (Figure S1). Sample missing rate significantly varied by batch; seven batches were considered outliers based on mean missing rates exceeding the third quartile plus 1.5 times the interquartile range (Figure S2). After excluding sex mismatches, a total of 588 samples remained, including 571 trial participants (10 genotyped twice) and seven HapMap samples. We advise researchers using the data to adjust genetic models for specimen type and study site and consider a sensitivity analysis excluding batch outliers.

### 3.3. Ancestry

**Figure 2:**
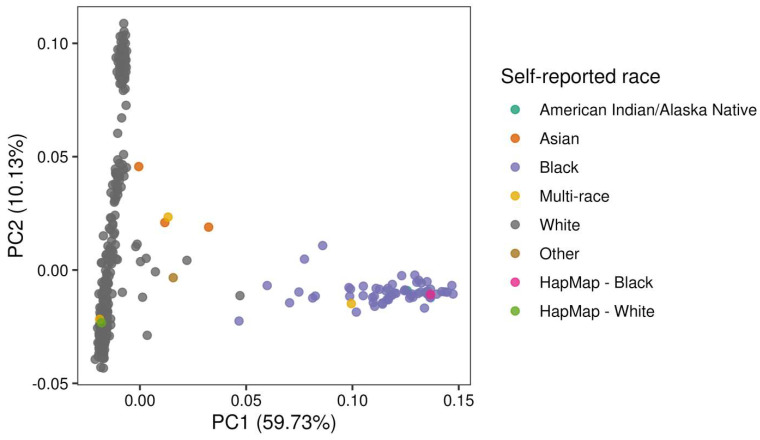
Principal component analysis (PC1 vs. PC2) of genetic data, annotated by self-reported ancestry. Each dot represents an individual (trial participants or HapMap samples), colored according to their self-reported ancestry group. The percentage of variance explained by each PC was annotated on the corresponding axis.

A preliminary PCA used unrelated samples (568 trial participants, two unique HapMap) and a subset of independent autosomal biallelic SNPs (n = 74,816). For each sibling or expected duplicate pair, we selected the sample with the higher SNP call rate. The first, second, and third PC explained 59.7%, 10.1%, and 4.7% of the variation, respectively (Figure 2). PC1 clearly separated White participants from Black participants. The three Asian participants and one ‘other’ participant clustered closer to White participants than Black participants, while the ‘multi-race’ participants were not discernable. We used PC1 to define a subgroup of European-ancestry trial participants; defined as all self-identified White participants falling within six SD of the means of PC1 for this subgroup (N = 499); two self-identified White participants were outliers. A smaller subgroup of African-ancestry participants was defined using the same criteria (N = 59); no outliers were detected. The ancestry of participants of other self-identified races (N = 8) could not be confidently inferred, and thus, their genetic data were not processed further or included in the final genetic dataset. A second pass kinship analysis did not yield any additional related participants of 2nd-degree or closer (all kinship coefficients < 0.0884).

### 3.4. SNP QC: Genotype Concordance

We evaluated genotype concordance using seven duplicate sample pairs with inferred European ancestry and sample call rates > 95% for both samples. Across pairs, concordance rates ranged from 98.1% to 99.8%, indicating high overall genotype consistency. A total of 34,164 SNPs showed discordance in at least one pair, with 8297 SNPs discordant in more than one pair. Only a single SNP was discordant across all seven pairs. The number of discordant SNPs ranged from 1344 to 12,818 across pairs (Figure S3A). More discordant SNPs were observed among serum–serum pairs than blood–serum pairs (Figure S3B). We did not exclude discordant SNPs from the genetic dataset but provide the pooled list to inform downstream analysis.

### 3.5. Sample QC2: Runs of Homozygosity

**Figure 3:**
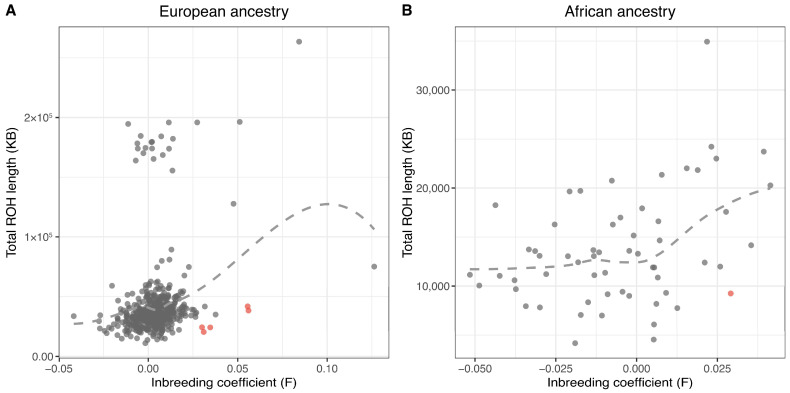
Identification of sample outliers based on heterozygosity and runs of homozygosity (ROH). (**A**) Among 499 trial samples of inferred European ancestry, five samples (in red) with unusually short ROH relative to their heterozygosity were flagged as outliers based on residuals from a LOESS regression. (**B**) Among 59 African ancestry samples, one outlier (in red) was identified using the same approach.

We assessed heterozygosity and ROH to identify potential low-quality samples across ancestry groups. Among 499 trial samples of inferred European ancestry, five samples (1.0%) had unusually short ROH relative to their heterozygosity and were flagged as outliers based on residuals from a LOESS regression of ROH on heterozygosity (Figure 3A). Similarly, among 59 African ancestry trial samples, one sample (1.7%) was flagged as an outlier using the same approach (Figure 3B). These outliers were excluded from downstream analysis.

### 3.6. Population Substructure

After sample QC, a total of 494 and 58 unrelated trial samples of inferred European and African ancestry, respectively, was retained. A per-ancestry PCA was conducted using subsets of independent autosomal biallelic SNPs with call rates > 98%, MAF > 5%, and HWE *p* > 1 × 10^−6^ (n = 67,046 for European ancestry and n = 76,188 for African ancestry). Population substructure remained evident within the European ancestry group (Figures S4 and S5). We provide and recommend adjusting for the top 10 genetic PCs in downstream analyses to account for population stratification.

### 3.7. Final QC Dataset and SNP Imputation

**Figure 4:**
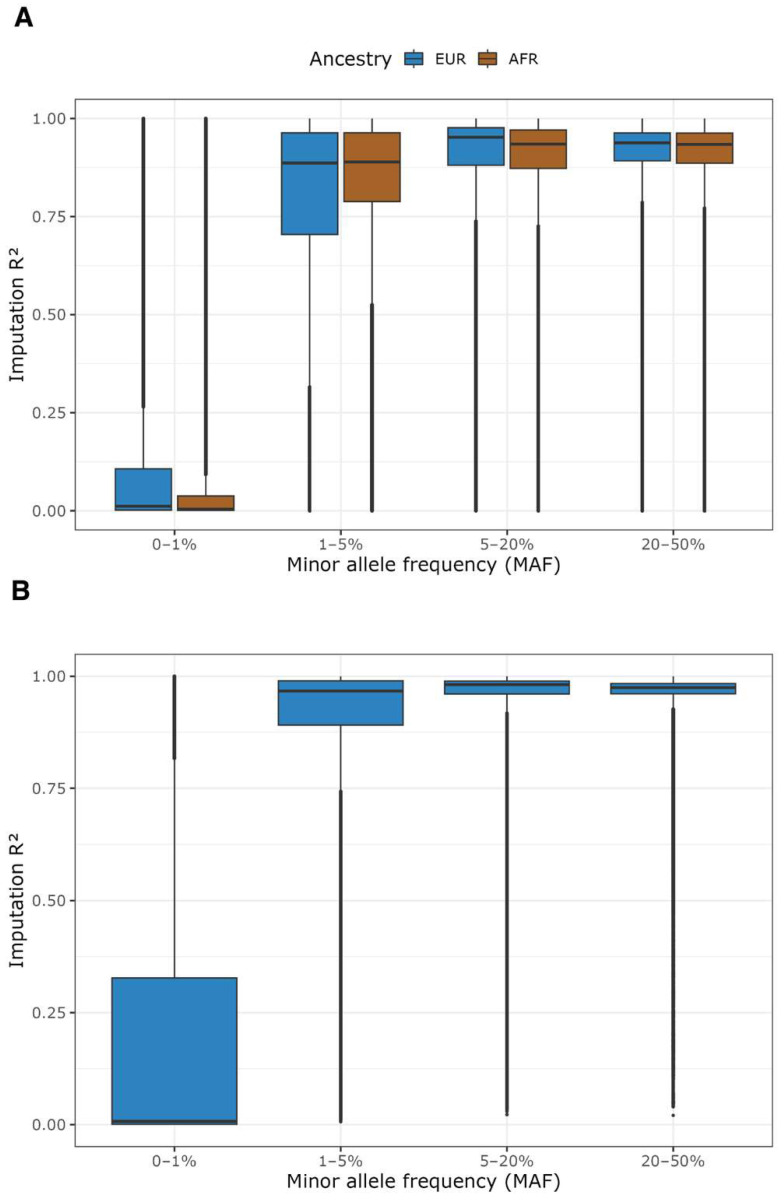
Imputation R^2^ using the 1000 Genomes Phase 3 v5 (1000G) and Haplotype Reference Consortium (HRC) reference panels. (**A**) Imputation R^2^ across minor allele frequency (MAF) bins using the 1000G reference panel in individuals of European (EUR) and African (AFR) ancestry. (**B**) Imputation R^2^ across MAF bins using the HRC reference panel in individuals of European (EUR) ancestry.

One of each sibling pair and expected duplicate pair was removed from the final genetic dataset (see Section 3.2). Thus, after all sample and SNP QC procedures, a total of 809,442 variants with call rates > 95%, MAF > 0.01, and HWE *p* > 1 × 10^−6^ were retained for 494 MIND trial participants of inferred European ancestry. For 58 participants of inferred African ancestry, 772,662 variants meeting the same criteria were retained. Characteristics of these participants are presented in Table 1. Following imputation using the 1000G reference panel, approximately 47.1 million variants were obtained for each ancestry group. Imputation quality was highest among common variants (MAF ≥ 5%), with 98.8% and 99.3% of variants achieving an imputation R^2^ ≥ 0.3, and 88.2% and 89.2% achieving an imputation R^2^ ≥ 0.8, in European and African ancestry groups, respectively; the imputation quality was lower for low-frequency and rare variants (Figure 4A and Table S1). HRC imputation was performed only for individuals of European ancestry, resulting in approximately 39.1 million variants. Compared to 1000G, HRC showed higher imputation quality for common and low-frequency variants, with 99.9% of common variants and 98.2% of low-frequency variants achieving an imputation R^2^ ≥ 0.3, but lower quality for rare variants (Figure 4B and Table S2). No post-imputation variant filtering based on imputation R^2^ or MAF was applied at this stage; variants should be filtered based on imputation quality thresholds (e.g., imputation R^2^ ≥ 0.3 and MAF > 0.01) as appropriate in downstream analyses.

### 3.8. Validity of Genetic Data

SNPs rs429358 and rs7412 in *APOE* were imputed using the 1000G reference panel with imputation R^2^ of 0.98 and 0.99, respectively, for the European ancestry sample, and 0.95 and 0.97 for the African ancestry sample. *APOE* genotype concordance of sequenced and imputed genotypes was 98.2% (Table S3). Assuming sequenced genotype the gold standard, the imputed genotypes misclassified two E4-carriers as non-carriers and five non-carriers as E4-carriers. Using the HRC panel, both SNPs were imputed with R^2^ values of 0.99 in the European ancestry sample and yielded *APOE* genotypes identical to that of 1000G. Replication of prior loci was restricted to MIND trial participants of European ancestry. Imputation quality measures are presented in Table S4. Sample sizes ranged from 355 (fatty acids) to 494 (self-reported diet, BMI) across selected traits and were much smaller than those reported in corresponding published genome-wide association studies (GWAS). We replicated at least one SNP previously associated with adiponectin (rs16861209, *p* = 1.5 × 10^−5^), ALA (rs174547, *p* = 1.3 × 10^−7^), alpha-tocopherol (rs964184, *p* = 0.003), B12 (rs602662, *p* = 0.02), beta-carotene (*p* = 0.0002), coffee intake (rs4410790, *p* = 0.04), tea intake (rs2472297, *p* = 0.047), and DHA (rs174528, *p* = 0.01) in the 1000G-imputed data (Table S5). Similar results were observed using the HRC-imputed data. We did not replicate SNP associations with other traits tested.

## 4. Discussion

The current study describes the genetic data and QC pipeline for the first RCT of a calorie-restricted MIND diet on cognitive change [5]. No significant differences in the primary endpoint, cognitive change, after 3 years were observed compared to a calorie-restricted control diet. It is possible the repeated cognitive testing that occurred in both the intervention and control group improved test performance or the duration of the trial was insufficient to parse benefit of the MIND diet over control [17]. Genetic differences in age-related cognitive decline or in metabolism of constituents characteristic of MIND might also enhance or limit efficacy and ultimately impact response to this diet. This genetic resource serves to address this variation in secondary analysis of the MIND RCT and will be made available to researchers via The National Institute on Aging Genetics of Alzheimer’s Disease Data Storage Site (NIAGADS, https://dss.niagads.org/ (accessed on 1 July 2025). Specifically, ancestry-specific (European, African) cleaned raw genotyping and unfiltered imputation files, sample meta files including study site, specimen, batch, batch outlier, and PCs, and discordant SNP lists will be made available.

Blood buffy coats are generally the preferred specimens for DNA. Many biobanks, cohorts, and completed trials archive serum samples, a fraction of blood that does not include blood cells and clotting factors but still may include cellular debris and low amounts of circulating DNA. Consistent with other reports [18,19], we demonstrated adequacy of serum as an alternate DNA source and thus potential to activate archived serum samples for array-based research. Although serum DNA extraction and genotyping performance metrics were inferior to those for blood, the option to use serum is attractive in cases of insufficient or missing whole bloods.

Imputation using the 1000G and the HRC, two widely used and complementary reference panels, yielded high-quality imputed data for both European and African ancestry populations. The 1000G includes a broad range of global populations and provides better coverage of rare and ancestry-specific variants, particularly for non-European groups, which enabled us to perform high-quality imputation for individuals of African ancestry. In contrast, the HRC panel, with its large sample size and dense coverage of common variants in European populations, enables more accurate imputation of low-frequency and common variants in the European ancestry population. Researchers using these data can choose between the reference panels based on their analytic needs. We further confirmed high concordance between imputed and sequencing-based *APOE* genotypes, supporting the reliability of our genotyping and imputation pipeline as well as the feasibility of using serum-derived DNA. To validate the quality of the genetic data more broadly, we successfully replicated several known GWAS associations with diet-related biomarkers (e.g., adiponectin, alpha-linolenic acid, alpha-tocopherol), despite limited statistical power for GWAS discovery in this trial-sized cohort.

While prior studies have explored *APOE* ε4 as a potential modifier of dietary associations with cognitive outcomes, findings have been mixed, with some studies reporting differential associations and others, including the MIND trial and UK Biobank, reporting null interactions [5,20,21,22,23,24]. Rather than focusing solely on *APOE*, the genome-wide genetic data in our trial open the door for more comprehensive analyses of the genetic contributions to variability in dietary response, molecular profiles, and cognitive outcomes. Clinical, imaging, and biological markers of cognition and aging have been collected over the duration of the trial [6]. A battery of 12 cognitive tests was administered during clinical visits at baseline, 6 months, 12 months, 24 months, and 36 months. The test battery included multiple tests for each of four cognitive domains, including episodic memory (immediate and delayed recall of the East Boston story, CERAD word list learning, recall, and recognition), semantic memory (category fluency, Multilingual Naming Test), executive function (Trails B, NIH toolbox flanker test), and perceptual speed (Trails A, Digit Symbol Substitution Test, NIH toolbox pattern comparison test). A total of 267 participants agreed to undergo brain imaging at baseline, 201 of whom underwent follow-up imaging at 36 months [5]. Existing MRI-derived measures include total brain volume, hippocampal volume, white/gray matter volumes, segmented gray matter regions, white matter lesions, and thickness of segmented cortical regions. About 508 participants have measures of established AD biomarkers, including pTau-181, neurofilament light chain, and glial fibrillary acidic protein at baseline and 36 months. We plan to use this genetic resource to investigate whether changes in clinical, imaging, and biological markers of cognition in response to MIND are modified by genetic differences in AD susceptibility and nutrient metabolism. The former would extend our *APOE*–MIND interaction analysis [5] to a more comprehensive score of AD susceptibility, and the latter may yield insight into specific components of the MIND diet (i.e., folate, vitamin K, carotenoids) that mediate response to the diet. Weight, 24-hour dietary recalls, and blood and urine biomarkers of diet (Table S5) were measured at baseline and over the course of follow-up. The microbiome was profiled from fecal samples collected from 257 participants. Thus, baseline and change in weight, nutrient biomarkers, and microbiome in response to diet may also be subject to genetic analysis. The availability of genome-wide data in a randomized trial setting provides new opportunities to conduct systems-level analyses to explore how genetic variation may influence responses to dietary interventions and shape downstream omics profiles. The trial design also offers a strong basis for causal interpretation, ultimately informing more targeted approaches to cognitive health and precision nutrition.

Our study has several limitations. Although we demonstrated that serum is a viable alternative to whole blood for DNA extraction and genotyping with comparable performance, potential batch effects related to specimen type remain. We recommend adjusting for specimen type as a covariate in downstream analyses. Minor batch effects related to study site or genotyping array batch may also be present; researchers should consider adjusting for study site and excluding array batch outliers as sensitivity analyses. Our study population was predominantly of European ancestry, and individuals with uncertain or unclassifiable ancestry were excluded, which may limit the generalizability of downstream findings. Nevertheless, the inclusion of a subset of individuals of African ancestry with high-quality imputation offers a valuable opportunity to investigate potential differences in dietary intervention response across genetic backgrounds. Investigators interested in using this resource outside the context of the RCT will also need to consider the inclusion and exclusion factors. All participants were at least 65 years of age, cognitively unimpaired, overweight, and had a family history of dementia and a suboptimal diet.

## 5. Conclusions

In summary, we present the first genetic resource derived from a randomized controlled trial of a dietary intervention targeting cognitive outcomes, providing a valuable foundation for gene–diet interaction studies, ancestry-specific analyses, and integrative multi-omics research aimed at advancing precision nutrition and promoting cognitive health.

## Figures and Tables

**Table 1:** Baseline characteristics of MIND trial participants with high-quality genetic data.

Characteristic	EUR (N = 494)	AFR (N = 58)
Age, years	69.9 (4.2)	69.4 (4.0)
Female, %	64	81
At least college degree, %	79	43
BMI	33.6 (5.6)	35.9 (6.7)
Current smoker, %	2	7
Diabetes, %	14	28
MIND score	7.7 (1.8)	7.9 (1.7)
Study Site, %		
Rush	48	76
Harvard	52	24
*APOE* ℇ4-carrier, %	28	29
DNA specimen, %		
blood	66	53
serum	34	47

AFR: African ancestry; BMI: body mass index; EUR: European ancestry; MIND: Mediterranean-DASH Intervention for Neurodegenerative Delay.

## Data Availability

Cleaned genotyping files, imputed files, and corresponding meta-data (batch outliers, PCs, DNA specimen, sex) described in the current study will be made available via NIAGADS, https://dss.niagads.org/, in accordance with NIH/NIA guidelines for genetic resource sharing. MIND-Trial phenotyping can be accessed upon approval at https://www.radc.rush.edu (accessed on 1 July 2025).

## Supplementary Materials

The following supporting information can be downloaded at: https://www.mdpi.com/article/10.3390/nu17152548/s1, Figure S1. Sample missing rate by study site (Rush, Harvard) stratified by specimen type (blood, serum). Figure S2. Sample missing rate by genotyping array batch. Figure S3. Genotype concordance between duplicate pairs. Figure S4. Principal component analysis (PC1-PC10) of post-quality control genetic data among samples of inferred European ancestry. Figure S5. Principal component analysis (PC1-PC10) of post-quality control genetic data among samples of inferred African ancestry. Table S1. Imputation R^2^ using the 1000 Genomes Phase 3 v5 (1000G) reference panel. Table S2. Imputation R^2^ using the Haplotype Reference Consortium (HRC) r1.1 reference panel. Table S3. *APOE* genotype concordance using the 1000G and HRC reference panels. Table S4. Published GWAS loci and imputation quality measures in MIND genetic data. Table S5. Replication of previously published GWAS loci in MIND genetic data (EUR, 1000G-imputed).
