# Supplementary material for: Mediterranean-DASH Intervention for Neurodegenerative Delay (MIND) Trial: Genetic Resource for Precision Nutrition
Source: Nutrients. 2025 Aug 4;17(15):2548. doi: 10.3390/nu17152548 (PMC12348084; doi:10.3390/nu17152548)
Supplement: Supplementary file 1 [file nutrients-17-02548-s001.zip › nutrients-3756506-supplementary.pdf]

Supplementary Materials

Mediterranean-DASH Intervention for Neurodegenerative Delay (MIND) Trial: Genetic Resource for Precision Nutrition

Yuxi Liu, Hailie Fowler, Dong D. Wang, Lisa L. Barnes, Marilyn C. Cornelis

Supplementary Figure Legends

Figure S1. Sample missing rate by study site (Rush, Harvard) stratified by specimen type (blood, serum).

Figure S2. Sample missing rate by genotyping array batch. Batches with mean missing rates exceeding the third quartile plus 1.5 times the interquartile range of missing rates across all batches were considered outliers and are highlighted in red.

Figure S3. Genotype concordance between duplicate pairs. A, Number of SNP mismatches across all seven duplicate pairs. **B,** Number of SNP mismatches by type of specimen pairs (blood-serum, serum-serum).

Figure S4. Principal component analysis (PC1-PC10) of post-quality control genetic data among samples of inferred European ancestry. Each dot represents an individual, with the percentage of variance explained by each PC indicated on the corresponding axis.

Figure S5. Principal component analysis (PC1-PC10) of post-quality control genetic data among samples of inferred African ancestry. Each dot represents an individual, with the percentage of variance explained by each PC indicated on the corresponding axis.

Table S1. Imputation R^2 using the 1000 Genomes Phase 3 v5 (1000G) reference panel

Ancestry	MAF bin	Total N	Mean R^2*	$R^2 \geq 0.3$	$R^2 \geq 0.5$	$R^2 \geq 0.8$	$R^2 \geq 0.9$
EUR	<1%	36979387 (78.5%)	0.14	6399594 (17.3%)	4772372 (12.9%)	2037634 (5.5%)	1084657 (2.9%)
	1-5%	3033120 (6.4%)	0.78	2747351 (90.6%)	2617824 (86.3%)	1962061 (64.7%)	1421516 (46.9%)
	≥5%	7096962 (15.1%)	0.90	7013289 (98.8%)	6927737 (97.6%)	6262335 (88.2%)	5084572 (71.6%)
	Total	47109469 (100%)	0.30	16160234 (34.3%)	14317933 (30.4%)	10262030 (21.8%)	7590745 (16.1%)
AFR	<1%	31060929 (65.9%)	0.12	3980648 (12.8%)	3254220 (10.5%)	2518829 (8.1%)	2053814 (6.6%)
	1-5%	6428434 (13.6%)	0.84	6297425 (98.0%)	6135983 (95.5%)	4678946 (72.8%)	3000211 (46.7%)
	≥5%	9620105 (20.4%)	0.90	9554537 (99.3%)	9486672 (98.6%)	8577051 (89.2%)	6515411 (67.7%)
	Total	47109468 (100%)	0.38	19832610 (42.1%)	18876875 (40.1%)	15774826 (33.5%)	11569436 (24.6%)

AFR: African ancestry; EUR: European ancestry; MAF: minor allele frequency
*R^2: Estimated squared correlation between imputed and true genotype dosages

Table S2. Imputation R^2 using the Haplotype Reference Consortium (HRC) r1.1 reference panel

Ancestry	MAF bin	Total N	Mean R^2*	$R^2 \geq 0.3$	$R^2 \geq 0.5$	$R^2 \geq 0.8$	$R^2 \geq 0.9$
EUR	<1%	31239496 (79.9%)	0.22	7932493 (25.4%)	7112902 (22.8%)	6078065 (19.5%)	5064210 (16.2%)
	1-5%	2401592 (6.1%)	0.91	2357356 (98.2%)	2331753 (97.1%)	2262810 (94.2%)	2128910 (88.6%)
	≥5%	5476017 (14.0%)	0.96	5470156 (99.9%)	5463107 (99.8%)	5442781 (99.4%)	5401590 (98.6%)
	Total	39117105 (100%)	0.37	15760005 (40.3%)	14907762 (38.1%)	13783656 (35.2%)	12594710 (32.2%)

EUR: European ancestry; MAF: minor allele frequency

*R^2: Estimated squared correlation between imputed and true genotype dosages

Table S3. *APOE* genotype concordance using the 1000G and HRC reference panels

Sequenced genotype	Imputed genotype					
	E2/E2	E2/E3	E2/E4	E3/E3	E3/E4	E4/E4
E2/E2	4	0	0	0	0	0
E2/E3	0	71	0	2	0	0
E2/E4	0	0	12	0	0	0
E3/E3	0	0	0	314	**5**	0
E3/E4	0	**1**	0	**1**	129	0
E4/E4	0	0	0	0	1	12

APOE4 genotype was derived from SNPs rs429358 and rs7412, with identical grouping using both the 1000G and HRC reference panels.

Table S4. Published GWAS loci and imputation quality measures in MIND genetic data

Trait	PMID	Chr	Pos	SNP	A1	A2	EUR 1000G-imputed					EUR HRC-imputed					AFR 1000G-imputed				
							A1 Freq	SNP Type*	AVG CS†	R^2‡	ER^2§	A1 Freq	SNP Type*	AVG CS†	R^2‡	ER^2§	A1 Freq	SNP Type*	AVG CS†	R^2‡	ER^2§
adiponectin	37859345	3	186563114	rs16861209	A	C	0.103	I	0.998	0.983	N/A	0.103	I	0.998	0.984	NA	0.123	I	0.996	0.967	NA
adiponectin	37859345	16	81534790	rs2925979	T	C	0.265	T/I	1.000	0.999	0.957	0.265	T/I	1.000	1.000	0.987	0.293	T/I	1.000	0.999	0.912
adiponectin	37859345	3	52720080	rs1108842	A	C	0.487	T/I	1.000	1.000	0.999	0.487	T/I	1.000	1.000	0.999	0.474	T/I	1.000	1.000	1.000
adiponectin	37859345	16	82663288	rs12051272	T	G	0.011	T/I	1.000	0.999	0.913	0.011	T/I	1.000	1.000	1.000	0.034	T/I	1.000	1.000	1.000
adiponectin	37859345	19	33899065	rs731839	G	A	0.360	T/I	1.000	1.000	0.991	0.360	T/I	1.000	1.000	0.995	0.509	T/I	1.000	1.000	0.957
alpha-linolenic acid	21829377	11	61570783	rs174547	C	T	0.330	T/I	0.999	0.998	0.986	0.315	T/I	1.000	1.000	1.000	0.103	T/I	1.000	1.000	1.000
alpha-linolenic acid	21829377	11	61417472	rs1692120	G	A	0.495	T/I	1.000	1.000	0.984	0.495	T/I	1.000	1.000	1.000	0.490	T/I	0.998	0.995	0.881
alpha carotene	28002826	1	223872051	rs12137025	C	T	0.074	I	0.999	0.980	N/A	0.076	I	0.999	0.985	NA	0.050	I	0.996	0.933	NA
alpha carotene	28002826	4	182178306	rs17830069	G	A	0.045	I	0.999	0.984	N/A	0.045	I	1.000	0.995	NA	0.037	I	0.996	0.929	NA
alpha carotene	28002826	2	46409466	rs2594495	A	G	0.127	I	0.984	0.892	N/A	0.124	I	0.996	0.969	NA	0.193	I	0.981	0.905	NA
alpha tocopherol	23696881	11	116648917	rs964184	G	C	0.158	T/I	1.000	1.000	1.000	0.158	T/I	1.000	1.000	1.000	0.207	T/I	1.000	1.000	1.000
alpha tocopherol	23696881	19	19379549	rs58542926	T	C	0.065	T/I	1.000	1.000	0.970	0.065	T/I	1.000	1.000	0.996	0.060	T/I	1.000	0.998	0.828
B12	23754956	19	49206985	rs602662	A	G	0.498	T/I	1.000	1.000	0.999	0.498	T/I	1.000	1.000	1.000	0.466	T/I	1.000	1.000	1.000
B12	23754956	10	17156151	rs1801222	A	G	0.318	T/I	0.999	0.998	0.968	0.318	T/I	1.000	0.998	0.974	0.162	T/I	0.998	0.989	0.822
B12	23754956	11	59623378	rs34324219	A	C	0.129	T/I	1.000	1.000	0.906	0.129	T/I	1.000	1.000	0.964	0.078	T/I	1.000	1.000	0.923
B12	23754956	19	8367709	rs2336573	T	C	0.056	I	0.987	0.765	N/A	0.051	I	0.997	0.948	NA	0.259	T/I	1.000	1.000	0.954
B12	23754956	22	31018975	rs1131603	C	T	0.055	T/I	1.000	0.998	0.947	0.055	T/I	1.000	0.999	0.938	0.004	I	0.996	0.182	NA
B12	23754956	14	74759006	rs3742801	T	C	0.376	T/I	1.000	1.000	0.975	0.376	T/I	1.000	1.000	0.990	0.241	T/I	1.000	1.000	0.892
B12	23754956	6	49412433	rs1141321	T	C	0.358	T/I	1.000	1.000	1.000	0.358	T/I	1.000	1.000	1.000	0.268	T/I	1.000	0.998	0.956
B12	23754956	4	146576418	rs2270655	C	G	0.050	T/I	1.000	0.999	0.962	0.050	T/I	1.000	1.000	0.995	0.026	T/I	1.000	1.000	0.842
B12	23754956	13	100518634	rs41281112	T	C	0.032	T/I	1.000	0.998	0.653	0.032	T/I	1.000	1.000	0.958	0.011	I	0.994	0.520	NA
beta carotene	23696881	16	81258987	rs12926540	C	T	0.490	I	0.974	0.910	N/A	0.498	I	0.990	0.965	NA	0.373	I	0.953	0.833	NA
BMI	36581621	18	58039276	rs2229616	T	C	0.015	T/I	1.000	1.000	1.000	0.015	T/I	1.000	1.000	1.000	0.019	I	0.998	0.922	NA
BMI	36581621	19	46181392	rs1800437	C	G	0.197	T/I	1.000	1.000	1.000	0.197	T/I	1.000	1.000	1.000	0.112	T/I	1.000	1.000	1.000

BMI	36581621	16	53877592	rs6499653	T	C	0.274	I	0.976	0.896	N/A	0.261	I	0.988	0.948	NA	0.451	I	0.981	0.937	NA
BMI	36581621	4	103188709	rs13107325	T	C	0.088	T/I	0.999	0.991	0.878	0.087	T/I	0.999	0.993	0.927	0.011	I	0.997	0.796	NA
BMI	36581621	4	25408838	rs34811474	A	G	0.227	T/I	1.000	0.999	0.895	0.226	T/I	1.000	0.999	0.920	0.052	T/I	1.000	0.998	0.650
BMI	36581621	12	50247468	rs7138803	A	G	0.441	T/I	1.000	1.000	0.996	0.441	T/I	1.000	1.000	0.991	0.172	T/I	1.000	1.000	0.996
BMI	36581621	9	28414339	rs10968576	G	A	0.283	T/I	1.000	1.000	1.000	0.283	T/I	1.000	1.000	1.000	0.190	T/I	1.000	1.000	0.958
BMI	36581621	1	78623626	rs17391694	T	C	0.098	T/I	1.000	0.999	0.912	0.099	T/I	1.000	0.997	0.937	0.034	T/I	1.000	0.999	0.750
BMI	36581621	18	57839769	rs571312	A	C	0.232	T/I	1.000	1.000	0.971	0.232	T/I	1.000	1.000	0.999	0.362	T/I	1.000	1.000	0.972
BMI	36581621	3	185834499	rs9816226	A	T	0.168	T/I	1.000	1.000	0.999	0.168	T/I	1.000	1.000	1.000	0.184	T/I	0.997	0.987	0.938
BMI	36581621	11	47650993	rs3817334	T	C	0.416	T/I	1.000	1.000	1.000	0.416	T/I	1.000	1.000	1.000	0.284	T/I	1.000	1.000	0.998
BMI	36581621	16	19933600	rs12444979	T	C	0.143	T/I	1.000	1.000	0.999	0.143	T/I	1.000	1.000	1.000	0.103	T/I	1.000	1.000	0.999
BMI	36581621	10	114758349	rs7903146	T	C	0.279	T/I	1.000	1.000	0.982	0.279	T/I	1.000	1.000	0.999	0.326	T/I	0.999	0.995	0.956
BMI	36581621	15	68086838	rs2241423	A	G	0.227	T/I	1.000	1.000	1.000	0.227	T/I	1.000	1.000	1.000	0.362	T/I	1.000	1.000	1.000
BMI	36581621	1	201860626	rs2250377	A	G	0.330	T/I	1.000	1.000	0.998	0.330	T/I	1.000	1.000	1.000	0.202	T/I	0.996	0.987	0.948
BMI	36581621	14	79945162	rs10146997	G	A	0.205	T/I	1.000	1.000	0.960	0.205	T/I	1.000	0.999	0.973	0.362	T/I	1.000	0.999	0.850
BMI	36581621	18	57942799	rs9956279	T	C	0.313	I	0.992	0.967	N/A	0.315	I	0.996	0.983	NA	0.107	I	0.994	0.949	NA
BMI	36581621	16	28885659	rs7359397	T	C	0.356	T/I	1.000	1.000	0.996	0.356	T/I	1.000	1.000	0.999	0.112	T/I	1.000	1.000	0.995
BMI	36581621	1	177889480	rs543874	G	A	0.197	T/I	1.000	1.000	1.000	0.197	T/I	1.000	1.000	1.000	0.267	T/I	1.000	1.000	0.990
BMI	36581621	19	47569003	rs3810291	G	A	0.320	T/I	0.999	0.998	0.947	0.323	T/I	1.000	0.999	0.962	0.776	I	0.962	0.802	NA
BMI	36581621	1	72765116	rs2568958	G	A	0.361	T/I	1.000	1.000	1.000	0.361	T/I	1.000	1.000	1.000	0.448	T/I	1.000	1.000	1.000
BMI	36581621	6	50820940	rs2635727	T	C	0.254	T/I	1.000	1.000	0.996	0.254	T/I	1.000	1.000	0.999	0.310	T/I	1.000	1.000	0.947
BMI	36581621	2	25141538	rs11676272	G	A	0.472	T/I	1.000	1.000	0.988	0.472	T/I	1.000	1.000	0.995	0.879	T/I	1.000	1.000	0.997
BMI	36581621	16	4015729	rs879620	C	T	0.422	T/I	0.999	0.999	0.982	0.422	T/I	1.000	0.999	0.989	0.734	T/I	0.997	0.989	0.800
BMI	36581621	11	27679916	rs6265	T	C	0.179	T/I	1.000	1.000	0.981	0.179	T/I	1.000	1.000	0.994	0.026	T/I	1.000	1.000	1.000
BMI	36581621	2	417167	rs62106258	C	T	0.057	I	0.996	0.941	N/A	0.057	I	0.998	0.966	NA	0.009	I	0.991	0.343	NA
BMI	36581621	2	634905	rs6548238	T	C	0.164	T/I	1.000	1.000	1.000	0.164	T/I	1.000	1.000	1.000	0.155	T/I	1.000	1.000	1.000
BMI	36581621	5	75015242	rs2112347	G	T	0.362	T/I	1.000	1.000	0.993	0.362	T/I	1.000	1.000	0.994	0.509	T/I	1.000	1.000	0.974
BMI	36581621	4	45182527	rs10938397	G	A	0.440	T/I	1.000	1.000	0.996	0.441	T/I	1.000	1.000	1.000	0.276	T/I	1.000	1.000	1.000
BMI	36581621	11	27583129	rs7481311	T	C	0.213	T/I	1.000	1.000	0.995	0.213	T/I	1.000	1.000	0.997	0.371	T/I	1.000	1.000	1.000

BMI	36581621	6	50803050	rs987237	G	A	0.188	T/I	1.000	1.000	0.996	0.188	T/I	1.000	1.000	0.995	0.095	T/I	1.000	1.000	1.000
caffeine/ coffee	25288136, 21490707	7	17284577	rs4410790	T	C	0.404	T/I	1.000	0.999	0.986	0.404	T/I	1.000	1.000	0.994	0.431	T/I	1.000	1.000	0.947
caffeine/ coffee	25288136, 21490707	15	75027880	rs2472297	T	C	0.198	T/I	1.000	0.999	0.925	0.200	T/I	0.999	0.997	0.927	0.060	T/I	1.000	1.000	0.999
relative carbohydrate intake measurement	34426670	16	73912588	rs1104608	C	G	0.432	I	0.972	0.909	N/A	0.432	I	0.988	0.959	NA	0.576	I	0.968	0.896	NA
relative carbohydrate intake measurement	34426670	19	49259529	rs838133	A	G	0.431	T/I	0.999	0.996	0.829	0.432	T/I	0.999	0.998	0.896	0.299	I	0.909	0.676	NA
relative carbohydrate intake measurement	34426670	8	9185146	rs2126259	T	C	0.090	T/I	1.000	1.000	1.000	0.090	T/I	1.000	1.000	1.000	0.147	T/I	1.000	1.000	1.000
relative carbohydrate intake measurement	34426670	3	25110415	rs7619139	T	A	0.460	I	0.964	0.875	N/A	0.441	I	0.987	0.953	NA	0.534	I	0.972	0.902	NA
coffee (drinker vs nondrinker)	25288136, 21490707	7	17284577	rs4410790	T	C	0.404	T/I	1.000	0.999	0.986	0.404	T/I	1.000	1.000	0.994	0.431	T/I	1.000	1.000	0.947
coffee (drinker vs nondrinker)	25288136, 21490707	15	75027880	rs2472297	T	C	0.198	T/I	1.000	0.999	0.925	0.200	T/I	0.999	0.997	0.927	0.060	T/I	1.000	1.000	0.999
docosahexaenoic acid	35213538	11	61543499	rs174528	C	T	0.352	T/I	1.000	0.999	0.974	0.353	T/I	1.000	0.999	0.985	0.440	T/I	1.000	1.000	0.991
docosahexaenoic acid	35213538	1	109817838	rs660240	T	C	0.220	T/I	1.000	1.000	1.000	0.220	T/I	1.000	1.000	0.999	0.319	T/I	1.000	1.000	1.000
docosahexaenoic acid	35213538	11	68562328	rs2229738	T	C	0.078	T/I	1.000	0.994	0.716	0.079	T/I	1.000	1.000	0.870	0.017	T/I	1.000	0.994	0.503
docosahexaenoic acid	35213538	15	58680178	rs261291	C	T	0.371	T/I	1.000	1.000	0.992	0.371	T/I	1.000	1.000	0.994	0.448	T/I	1.000	0.998	0.932
docosahexaenoic acid	35213538	8	126506694	rs112875651	A	G	0.367	I	0.987	0.951	N/A	0.367	I	0.992	0.969	NA	0.214	I	0.988	0.946	NA
docosahexaenoic acid	35213538	2	27730940	rs1260326	T	C	0.445	T/I	0.998	0.996	0.977	0.444	T/I	1.000	0.999	0.986	0.164	T/I	1.000	1.000	1.000
docosahexaenoic acid	35213538	2	21288321	rs562338	A	G	0.221	T/I	1.000	1.000	1.000	0.221	T/I	1.000	1.000	0.999	0.474	T/I	1.000	1.000	0.976
docosahexaenoic acid	35213538	16	56993324	rs3764261	A	C	0.313	T/I	1.000	1.000	1.000	0.313	T/I	1.000	1.000	1.000	0.354	T/I	0.999	0.996	0.963
docosahexaenoic acid	35213538	15	58723426	rs1077835	G	A	0.240	T/I	0.999	0.998	0.989	0.241	T/I	1.000	1.000	1.000	0.509	T/I	1.000	1.000	1.000
docosahexaenoic acid	35213538	1	62906489	rs638714	T	G	0.301	I	0.987	0.944	N/A	0.306	I	0.994	0.972	NA	0.682	I	0.989	0.949	NA
docosahexaenoic acid	35213538	10	65191645	rs7924036	T	G	0.490	I	0.979	0.923	N/A	0.500	I	0.991	0.968	NA	0.323	I	0.980	0.919	NA
docosahexaenoic acid	35213538	11	75450576	rs673335	C	T	0.169	I	0.992	0.945	N/A	0.166	I	0.997	0.979	NA	0.525	I	0.990	0.965	NA
docosahexaenoic acid	35213538	19	45413233	rs1065853	T	G	0.078	I	0.999	0.987	N/A	NA	NA	NA	NA	NA	0.121	I	0.997	0.971	NA

docosahexaenoic acid	35213538	18	47158234	rs9304381	C	T	0.155	T/I	0.999	0.995	0.977	0.157	T/I	1.000	0.999	0.969	0.371	T/I	1.000	1.000	1.000
docosahexaenoic acid	35213538	16	15127534	rs72789541	A	T	0.307	I	0.991	0.962	N/A	0.308	I	0.997	0.988	NA	0.223	I	0.995	0.971	NA
docosahexaenoic acid	35213538	15	58582540	rs1973688	T	C	0.296	I	0.997	0.984	N/A	0.298	I	0.997	0.988	NA	0.625	I	0.995	0.980	NA
docosahexaenoic acid	35213538	19	19379549	rs58542926	T	C	0.065	T/I	1.000	1.000	0.970	0.065	T/I	1.000	1.000	0.996	0.060	T/I	1.000	0.998	0.828
eicosapentaenoic acid	21829377	6	11008622	rs3798713	C	G	0.442	I	0.988	0.954	N/A	0.446	I	0.996	0.986	NA	0.241	T/I	1.000	1.000	1.000
eicosapentaenoic acid	21829377	11	61560081	rs174538	A	G	0.291	I	0.996	0.983	N/A	0.291	I	0.996	0.983	NA	0.103	I	0.998	0.983	NA
eicosapentaenoic acid	21829377	11	61722645	rs1109748	A	C	0.072	T/I	1.000	0.997	0.944	0.072	T/I	1.000	0.996	0.950	0.102	I	0.988	0.896	NA
eicosapentaenoic acid	21829377	11	61795586	rs11230874	G	T	0.026	I	0.997	0.879	N/A	0.025	I	0.999	0.957	NA	0.083	I	0.982	0.820	NA
relative fat intake measurement	34426670	2	25455389	rs752208	A	G	0.239	I	0.963	0.850	N/A	0.249	I	0.984	0.933	NA	0.277	I	0.941	0.787	NA
relative fat intake measurement	34426670	19	49259529	rs838133	A	G	0.431	T/I	0.999	0.996	0.829	0.432	T/I	0.999	0.998	0.896	0.299	I	0.909	0.676	NA
relative fat intake measurement	34426670	4	100239319	rs1229984	T	C	0.079	T/I	1.000	0.999	0.862	0.081	T/I	1.000	0.999	0.983	0.031	I	0.985	0.578	NA
Folate	23754956	1	11856378	rs1801133	A	G	0.367	T/I	1.000	0.999	0.766	0.369	T/I	1.000	0.998	0.867	0.112	T/I	1.000	0.997	0.465
Folate	23754956	11	71849741	rs652197	C	T	0.167	I	0.935	0.599	N/A	0.150	I	0.972	0.807	NA	0.605	I	0.940	0.801	NA
gamma tocopherol	36357675	22	30831852	rs11705639	A	G	0.195	I	0.990	0.944	N/A	0.195	I	0.997	0.983	NA	0.106	I	0.982	0.857	NA
gamma tocopherol	36357675	8	63883970	rs62508088	T	C	0.100	I	0.999	0.988	N/A	0.100	I	0.999	0.992	NA	0.047	I	0.996	0.939	NA
IL6	33517400	2	113841030	rs6734238	G	A	0.422	T/I	1.000	1.000	0.995	0.422	T/I	1.000	1.000	0.998	0.448	T/I	1.000	1.000	0.972
IL6	33517400	1	154630498	rs10796927	C	T	0.258	T/I	0.999	0.998	0.910	0.258	T/I	1.000	0.999	0.956	0.083	T/I	0.997	0.974	0.829
IL6	33517400	1	154430092	rs11265618	T	C	0.172	T/I	1.000	1.000	1.000	0.172	T/I	1.000	1.000	1.000	0.284	T/I	1.000	1.000	1.000
lycopene	26861389	4	140447105	rs7680948	C	A	0.266	I	0.983	0.928	N/A	0.264	I	0.993	0.970	NA	0.377	I	0.971	0.900	NA
relative protein intake measurement	34426670	3	25108179	rs1603977	T	A	0.364	I	0.964	0.866	N/A	0.347	I	0.988	0.950	NA	0.320	I	0.971	0.886	NA
relative protein intake measurement	34426670	2	27741237	rs780094	T	C	0.431	T/I	1.000	1.000	0.997	0.431	T/I	1.000	1.000	0.997	0.164	T/I	1.000	1.000	1.000
relative protein intake measurement	34426670	4	39413780	rs28712821	G	A	0.446	I	0.984	0.942	N/A	0.442	I	0.994	0.976	NA	0.467	I	0.972	0.912	NA
relative protein intake measurement	34426670	4	100239319	rs1229984	T	C	0.079	T/I	1.000	0.999	0.862	0.081	T/I	1.000	0.999	0.983	0.031	I	0.985	0.578	NA
relative protein intake measurement	34426670	19	49259529	rs838133	A	G	0.431	T/I	0.999	0.996	0.829	0.432	T/I	0.999	0.998	0.896	0.299	I	0.909	0.676	NA

retinol	36357675	10	95360964	rs10882283	C	A	0.373	I	0.939	0.800	N/A	0.376	I	0.975	0.914	NA	0.383	I	0.950	0.837	NA
retinol	36357675	18	29154045	rs1667237	C	G	0.480	I	0.989	0.958	N/A	0.364	I	0.996	0.983	NA	0.733	I	0.983	0.929	NA
retinol	36357675	2	27742603	rs780093	T	C	0.432	T/I	1.000	1.000	0.988	0.432	T/I	1.000	1.000	0.993	0.164	T/I	1.000	1.000	1.000
retinol	36357675	3	135926622	rs645040	G	T	0.212	T/I	1.000	1.000	1.000	0.212	T/I	1.000	1.000	1.000	0.224	T/I	1.000	1.000	1.000
tea (drinker vs nondrinker)	25288136, 21490707	15	75027880	rs2472297	T	C	0.198	T/I	1.000	0.999	0.925	0.200	T/I	0.999	0.997	0.927	0.060	T/I	1.000	1.000	0.999
tea (drinker vs nondrinker)	25288136, 21490707	7	17284577	rs4410790	T	C	0.404	T/I	1.000	0.999	0.986	0.404	T/I	1.000	1.000	0.994	0.431	T/I	1.000	1.000	0.947

AFR: African ancestry; AVG CS: average certainty score; A1: allele 1; A2: allele 2; Chr: chromosome; EUR: European ancestry; Freq: frequency; Pos: position

*SNP Type: imputed (I), typed (T)

†Average certainty score: Average of the maximum genotype probability across all samples for a variant

‡R^2: Estimated squared correlation between imputed and true genotype dosages

§ER^2: Empirical R^2 based on observed dosage variance

Table S5. Replication* of previously published GWAS loci in MIND genetic data (EUR, 1000G-imputed)

Trait	Published GWAS			MIND Trial Results								
	SNP-effect allele	Effect direction	PMID	N	Effect Allele	Effect allele frequency	Effect direction	*P*	SNP TYPE	Average certainty score†	R^2‡	ER^2§
adiponectin	rs16861209-A	increase	37859345	468	A	0.103	**increase**	**1.48E-05**	IMPUTED	0.998	0.983	N/A
adiponectin	rs2925979-C	increase	37859345	468	T	0.265	**decrease**	0.138142	TYPED/IMPUTED	1.000	0.999	0.957
adiponectin	rs1108842-C	increase	37859345	468	A	0.487	decrease	0.305536	TYPED/IMPUTED	1.000	1.000	0.999
adiponectin	rs12051272-G	decrease	37859345	468	T	0.011	decrease	0.454956	TYPED/IMPUTED	1.000	0.999	0.913
adiponectin	rs731839-A	increase	37859345	468	G	0.360	increase	0.807635	TYPED/IMPUTED	1.000	1.000	0.991
alpha-linolenic acid	rs174547-T	decrease	21829377	355	A	0.330	**increase**	**1.33E-07**	TYPED/IMPUTED	0.999	0.998	0.986
alpha-linolenic acid	rs1692120-A	decrease	21829377	355	G	0.495	decrease	0.832361	TYPED/IMPUTED	1.000	1.000	0.984
alpha carotene	rs12137025-C	increase	28002826	361	C	0.074	decrease	0.310795	IMPUTED	0.999	0.980	N/A
alpha carotene	rs17830069-G	increase	28002826	361	G	0.045	decrease	0.549321	IMPUTED	0.999	0.984	N/A
alpha carotene	rs2594495-A	increase	28002826	361	A	0.127	**increase**	0.750108	IMPUTED	0.984	0.892	N/A
alpha tocopherol	rs964184	NR	23696881	362	G	0.158	increase	**0.00342004**	TYPED/IMPUTED	1.000	1.000	1.000
alpha tocopherol	rs58542926	NR	23696881	362	T	0.065	decrease	**0.0113517**	TYPED/IMPUTED	1.000	1.000	0.970
B12	rs602662-A	increase	23754956	421	A	0.498	**increase**	**0.0222536**	TYPED/IMPUTED	1.000	1.000	0.999
B12	rs1801222-G	increase	23754956	421	A	0.318	**decrease**	0.118857	TYPED/IMPUTED	0.999	0.998	0.968
B12	rs34324219-C	increase	23754956	421	A	0.129	**decrease**	0.251322	TYPED/IMPUTED	1.000	1.000	0.906
B12	rs2336573-T	increase	23754956	421	T	0.056	**increase**	0.322438	IMPUTED	0.987	0.765	N/A
B12	rs1131603-C	increase	23754956	421	C	0.055	**increase**	0.52995	TYPED/IMPUTED	1.000	0.998	0.947
B12	rs3742801-T	increase	23754956	421	T	0.376	**increase**	0.602592	TYPED/IMPUTED	1.000	1.000	0.975
B12	rs1141321-C	increase	23754956	421	T	0.358	**decrease**	0.611267	TYPED/IMPUTED	1.000	1.000	1.000
B12	rs2270655-G	increase	23754956	421	C	0.050	increase	0.723694	TYPED/IMPUTED	1.000	0.999	0.962
B12	rs41281112-C	increase	23754956	421	T	0.032	**decrease**	0.869261	TYPED/IMPUTED	1.000	0.998	0.653
beta carotene	rs12926540-?	NR	23696881	361	C	0.490	increase (total)	**0.000244278**	IMPUTED	0.974	0.910	N/A
beta carotene	rs12926540-?	NR	23696881	361	C	0.490	increase (trans)	**0.000245812**	IMPUTED	0.974	0.910	N/A
beta carotene	rs12926540-?	NR	23696881	361	C	0.490	increase (cis)	**0.000403756**	IMPUTED	0.974	0.910	N/A
BMI	rs2229616-C	increase	36581621	494	T	0.015	increase	0.0735094	TYPED/IMPUTED	1.000	1.000	1.000

BMI	rs1800437-G	increase	36581621	494	C	0.197	increase	0.0957883	TYPED/IMPUTED	1.000	1.000	1.000
BMI	rs6499653-C	decrease	36581621	494	T	0.274	decrease	0.102193	IMPUTED	0.976	0.896	N/A
BMI	rs13107325-C	decrease	36581621	494	T	0.088	decrease	0.137486	TYPED/IMPUTED	0.999	0.991	0.878
BMI	rs34811474-G	increase	36581621	494	A	0.227	increase	0.155437	TYPED/IMPUTED	1.000	0.999	0.895
BMI	rs7138803-G	decrease	36581621	494	A	0.441	decrease	0.206168	TYPED/IMPUTED	1.000	1.000	0.996
BMI	rs10968576-G	increase	36581621	494	G	0.283	**increase**	0.235453	TYPED/IMPUTED	1.000	1.000	1.000
BMI	rs17391694-C	decrease	36581621	494	T	0.098	decrease	0.274362	TYPED/IMPUTED	1.000	0.999	0.912
BMI	rs571312-C	decrease	36581621	494	A	0.232	decrease	0.317183	TYPED/IMPUTED	1.000	1.000	0.971
BMI	rs9816226-T	increase	36581621	494	A	0.168	**decrease**	0.345969	TYPED/IMPUTED	1.000	1.000	0.999
BMI	rs3817334-C	decrease	36581621	494	T	0.416	**increase**	0.369857	TYPED/IMPUTED	1.000	1.000	1.000
BMI	rs12444979-C	increase	36581621	494	T	0.143	**decrease**	0.427007	TYPED/IMPUTED	1.000	1.000	0.999
BMI	rs7903146-C	increase	36581621	494	T	0.279	**decrease**	0.476965	TYPED/IMPUTED	1.000	1.000	0.982
BMI	rs2241423-G	increase	36581621	494	A	0.227	**decrease**	0.521148	TYPED/IMPUTED	1.000	1.000	1.000
BMI	rs2250377-G	decrease	36581621	494	A	0.330	decrease	0.529893	TYPED/IMPUTED	1.000	1.000	0.998
BMI	rs10146997-G	increase	36581621	494	G	0.205	decrease	0.543207	TYPED/IMPUTED	1.000	1.000	0.960
BMI	rs9956279-C	decrease	36581621	494	T	0.313	decrease	0.558657	IMPUTED	0.992	0.967	N/A
BMI	rs7359397-C	decrease	36581621	494	T	0.356	**increase**	0.646678	TYPED/IMPUTED	1.000	1.000	0.996
BMI	rs543874-G	increase	36581621	494	G	0.197	**increase**	0.653459	TYPED/IMPUTED	1.000	1.000	1.000
BMI	rs3810291-G	decrease	36581621	494	G	0.320	**decrease**	0.679009	TYPED/IMPUTED	0.999	0.998	0.947
BMI	rs2568958-G	decrease	36581621	494	G	0.361	increase	0.691768	TYPED/IMPUTED	1.000	1.000	1.000
BMI	rs2635727-C	increase	36581621	494	T	0.254	**decrease**	0.700922	TYPED/IMPUTED	1.000	1.000	0.996
BMI	rs11676272-G	increase	36581621	494	G	0.472	decrease	0.77103	TYPED/IMPUTED	1.000	1.000	0.988
BMI	rs879620-C	decrease	36581621	494	C	0.422	increase	0.788911	TYPED/IMPUTED	0.999	0.999	0.982
BMI	rs6265-C	increase	36581621	494	T	0.179	**decrease**	0.791503	TYPED/IMPUTED	1.000	1.000	0.981
BMI	rs62106258-C	decrease	36581621	494	C	0.057	increase	0.831901	IMPUTED	0.996	0.941	N/A
BMI	rs6548238-C	increase	36581621	494	T	0.164	increase	0.949311	TYPED/IMPUTED	1.000	1.000	1.000
BMI	rs2112347-G	decrease	36581621	494	G	0.362	**decrease**	0.960092	TYPED/IMPUTED	1.000	1.000	0.993
BMI	rs10938397-G	increase	36581621	494	G	0.440	**increase**	0.964979	TYPED/IMPUTED	1.000	1.000	0.996
BMI	rs7481311-C	decrease	36581621	494	T	0.213	decrease	0.967498	TYPED/IMPUTED	1.000	1.000	0.995
BMI	rs987237-G	increase	36581621	494	G	0.188	**increase**	0.977004	TYPED/IMPUTED	1.000	1.000	0.996

caffeine/coffee	rs4410790-T	decrease	25288136, 21490707	494	T	0.404	**decrease**	0.0872407	TYPED/IMPUTED	1.000	0.999	0.986
caffeine/coffee	rs2472297-T	increase	25288136, 21490707	494	T	0.198	**increase**	0.162321	TYPED/IMPUTED	1.000	0.999	0.925
relative carbohydrate intake measurement	rs1104608-C	increase	34426670	494	C	0.432	**increase**	0.228042	IMPUTED	0.972	0.909	N/A
relative carbohydrate intake measurement	rs838133-A	increase	34426670	494	A	0.431	decrease	0.304199	TYPED/IMPUTED	0.999	0.996	0.829
relative carbohydrate intake measurement	rs2126259-T	decrease	34426670	494	T	0.090	increase	0.329351	TYPED/IMPUTED	1.000	1.000	1.000
relative carbohydrate intake measurement	rs7619139-A	decrease	34426670	494	T	0.460	increase	0.641731	IMPUTED	0.964	0.875	N/A
coffee (drinker vs nondrinker)	rs4410790-T	decrease	25288136, 21490707	494	T	0.404	**decrease**	**0.0437819**	TYPED/IMPUTED	1.000	0.999	0.986
coffee (drinker vs nondrinker)	rs2472297-T	increase	25288136, 21490707	494	T	0.198	**increase**	0.479255	TYPED/IMPUTED	1.000	0.999	0.925
docosahexaenoic acid	rs174528-T	increase	35213538	355	C	0.352	**decrease**	**0.0129763**	TYPED/IMPUTED	1.000	0.999	0.974
docosahexaenoic acid	rs660240-T	decrease	35213538	355	T	0.220	**decrease**	**0.018207**	TYPED/IMPUTED	1.000	1.000	1.000
docosahexaenoic acid	rs2229738-C	increase	35213538	355	T	0.078	increase	0.0927063	TYPED/IMPUTED	1.000	0.994	0.716
docosahexaenoic acid	rs261291-T	decrease	35213538	355	C	0.371	decrease	0.194935	TYPED/IMPUTED	1.000	1.000	0.992
docosahexaenoic acid	rs112875651-G	increase	35213538	355	A	0.367	**decrease**	0.195687	IMPUTED	0.987	0.951	N/A
docosahexaenoic acid	rs1260326-T	increase	35213538	355	T	0.445	**increase**	0.229236	TYPED/IMPUTED	0.998	0.996	0.977
docosahexaenoic acid	rs562338-A	decrease	35213538	355	A	0.221	**decrease**	0.244093	TYPED/IMPUTED	1.000	1.000	1.000
docosahexaenoic acid	rs3764261-C	decrease	35213538	355	A	0.313	**increase**	0.535621	TYPED/IMPUTED	1.000	1.000	1.000
docosahexaenoic acid	rs1077835-A	decrease	35213538	355	G	0.240	**increase**	0.565029	TYPED/IMPUTED	0.999	0.998	0.989
docosahexaenoic acid	rs638714-G	increase	35213538	355	T	0.301	**decrease**	0.585258	IMPUTED	0.987	0.944	N/A
docosahexaenoic acid	rs7924036-G	decrease	35213538	355	T	0.490	**increase**	0.586474	IMPUTED	0.979	0.923	N/A
docosahexaenoic acid	rs673335-T	increase	35213538	355	C	0.169	**decrease**	0.666281	IMPUTED	0.992	0.945	N/A
docosahexaenoic acid	rs1065853-G	increase	35213538	355	T	0.078	increase	0.704241	IMPUTED	0.999	0.987	N/A
docosahexaenoic acid	rs9304381-C	decrease	35213538	355	C	0.155	**decrease**	0.846391	TYPED/IMPUTED	0.999	0.995	0.977
docosahexaenoic acid	rs72789541-T	increase	35213538	355	A	0.307	increase	0.858438	IMPUTED	0.991	0.962	N/A
docosahexaenoic acid	rs1973688-C	increase	35213538	355	T	0.296	increase	0.945874	IMPUTED	0.997	0.984	N/A
docosahexaenoic acid	rs58542926-C	increase	35213538	355	T	0.065	increase	0.951243	TYPED/IMPUTED	1.000	1.000	0.970
eicosapentaenoic acid	rs3798713-C	increase	21829377	355	C	0.442	**increase**	0.0605284	IMPUTED	0.988	0.954	N/A
eicosapentaenoic acid	rs174538-A	decrease	21829377	355	A	0.291	**decrease**	0.124468	IMPUTED	0.996	0.983	N/A
eicosapentaenoic acid	rs1109748-A	decrease	21829377	355	A	0.072	increase	0.787787	TYPED/IMPUTED	1.000	0.997	0.944
eicosapentaenoic acid	rs11230874-T	increase	21829377	355	G	0.026	**decrease**	0.818149	IMPUTED	0.997	0.879	N/A

relative fat intake measurement	rs752208-A	decrease	34426670	494	A	0.239	**decrease**	0.193459	IMPUTED	0.963	0.850	N/A
relative fat intake measurement	rs838133-A	decrease	34426670	494	A	0.431	increase	0.773797	TYPED/IMPUTED	0.999	0.996	0.829
relative fat intake measurement	rs1229984-T	increase	34426670	494	T	0.079	decrease	0.902338	TYPED/IMPUTED	1.000	0.999	0.862
Folate	rs1801133-G	increase	23754956	431	A	0.367	**decrease**	0.123554	TYPED/IMPUTED	1.000	0.999	0.766
Folate	rs652197-C	increase	23754956	431	C	0.167	**increase**	0.281744	IMPUTED	0.935	0.599	N/A
gamma tocopherol	rs11705639-A	increase	36357675	362	A	0.195	decrease	0.0736433	IMPUTED	0.990	0.944	N/A
gamma tocopherol	rs62508088-T	increase	36357675	362	T	0.100	decrease	0.233964	IMPUTED	0.999	0.988	N/A
IL6	rs6734238-G	increase	33517400	487	G	0.422	**increase**	0.383541	TYPED/IMPUTED	1.000	1.000	0.995
IL6	rs10796927-C	increase	33517400	487	C	0.258	decrease	0.787271	TYPED/IMPUTED	0.999	0.998	0.910
IL6	rs11265618-T	decrease	33517400	487	T	0.172	increase	0.837582	TYPED/IMPUTED	1.000	1.000	1.000
lycopene	rs7680948-A	decrease	26861389	361	C	0.266	**increase (trans)**	0.582805	IMPUTED	0.983	0.928	N/A
lycopene	rs7680948-A	decrease	26861389	361	C	0.266	**increase (total)**	0.730863	IMPUTED	0.983	0.928	N/A
lycopene	rs7680948-A	decrease	26861389	361	C	0.266	**increase (cis)**	0.910916	IMPUTED	0.983	0.928	N/A
relative protein intake measurement	rs1603977-A	increase	34426670	494	T	0.364	**decrease**	0.133819	IMPUTED	0.964	0.866	N/A
relative protein intake measurement	rs780094-T	increase	34426670	494	T	0.431	decrease	0.364013	TYPED/IMPUTED	1.000	1.000	0.997
relative protein intake measurement	rs28712821-T	increase	34426670	494	G	0.446	increase	0.49652	IMPUTED	0.984	0.942	N/A
relative protein intake measurement	rs1229984-A	decrease	34426670	494	T	0.079	decrease	0.610335	TYPED/IMPUTED	1.000	0.999	0.862
relative protein intake measurement	rs838133-A	decrease	34426670	494	A	0.431	increase	0.764525	TYPED/IMPUTED	0.999	0.996	0.829
retinol	rs10882283-A	increase	36357675	362	C	0.373	**decrease**	0.274015	IMPUTED	0.939	0.800	N/A
retinol	rs1667237-C	increase	36357675	362	A	0.480	**decrease**	0.560338	IMPUTED	0.989	0.958	N/A
retinol	rs780093-T	increase	36357675	362	T	0.432	**increase**	0.746529	TYPED/IMPUTED	1.000	1.000	0.988
retinol	rs645040-T	decrease	36357675	362	G	0.212	decrease	0.92599	TYPED/IMPUTED	1.000	1.000	1.000
tea (drinker vs nondrinker)	rs2472297-T	increase	25288136, 21490707	494	T	0.198	**increase**	**0.0465979**	TYPED/IMPUTED	1.000	0.999	0.925
tea (drinker vs nondrinker)	rs4410790-T	decrease	25288136, 21490707	494	T	0.404	increase	0.333197	TYPED/IMPUTED	1.000	0.999	0.986

*Defined as $P<0.05$ and directionally consistent with published GWAS.

†Average certainty score: Average of the maximum genotype probability across all samples for a variant

‡R^2: Estimated squared correlation between imputed and true genotype dosages
§ER^2: Empirical R^2 based on observed dosage variance

Figure S1

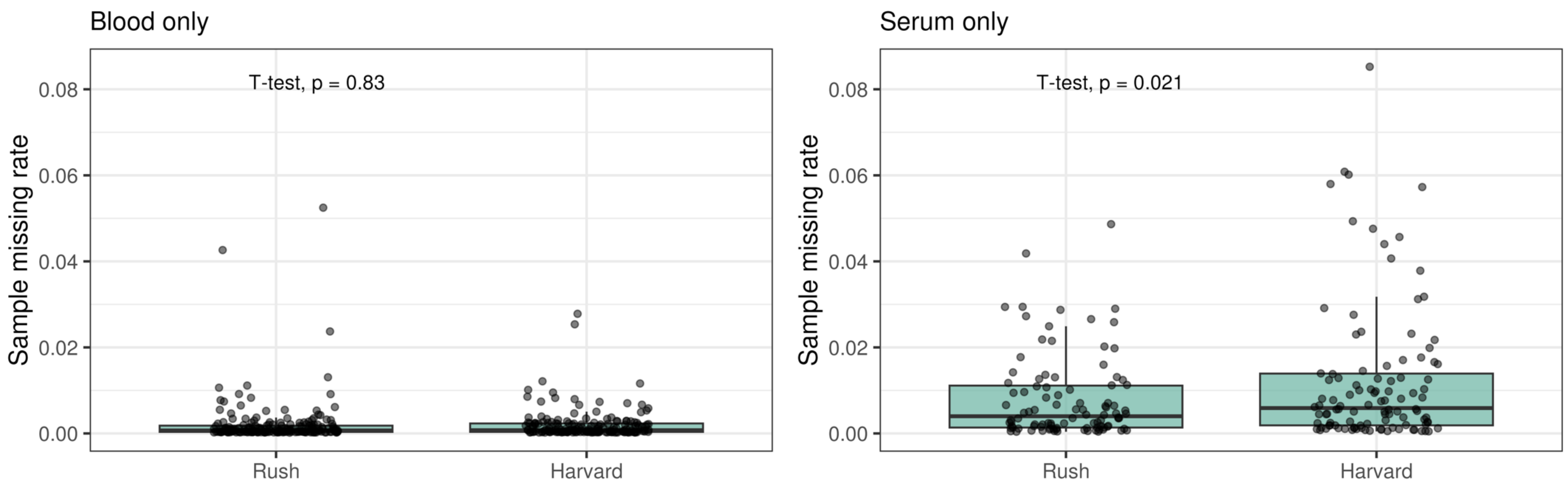

Blood only
T-test, p = 0.83
Sample missing rate
0.08
0.06
0.04
0.02
0.00
Rush
Harvard
Serum only
T-test, p = 0.021
Sample missing rate
0.08
0.06
0.04
0.02
0.00
Rush
Harvard

Figure S2

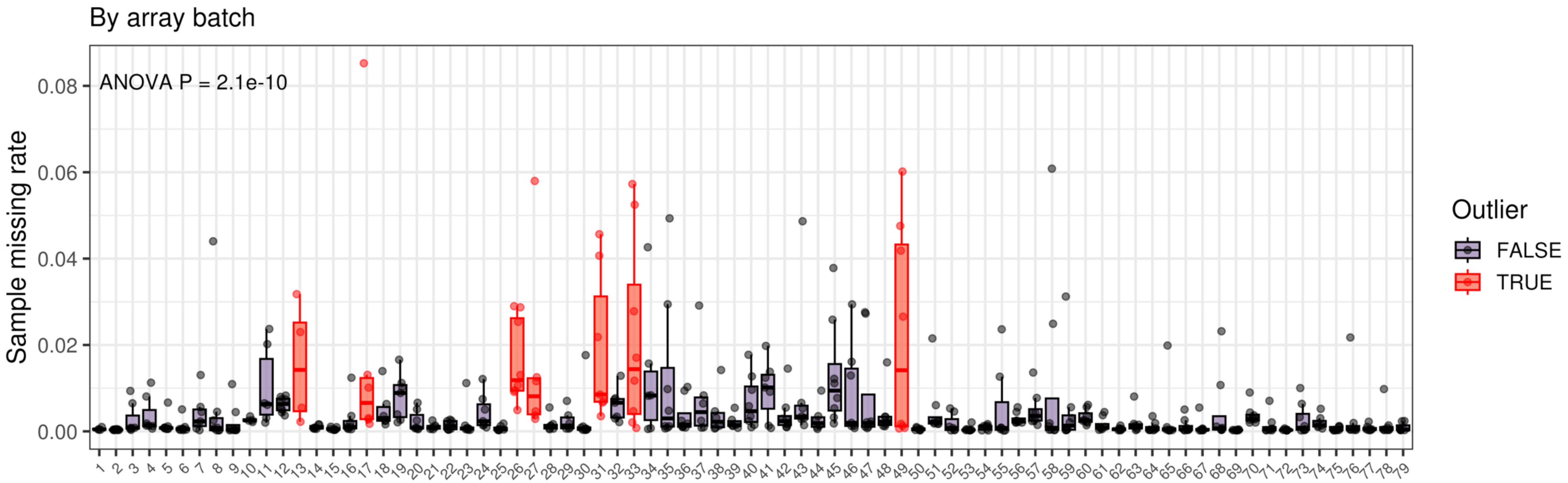
By array batch
ANOVA P = 2.1e-10
Sample missing rate
0.08
0.06
0.04
0.02
0.00
Outlier
FALSE
TRUE
1 2 3 4 5 6 7 8 9 10 11 12 13 14 15 16 17 18 19 20 21 22 23 24 25 26 27 28 29 30 31 32 33 34 35 36 37 38 39 40 41 42 43 44 45 46 47 48 49 50 51 52 53 54 55 56 57 58 59 60 61 62 63 64 65 66 67 68 69 70 71 72 73 74 75 76 77 78 79

Figure S3

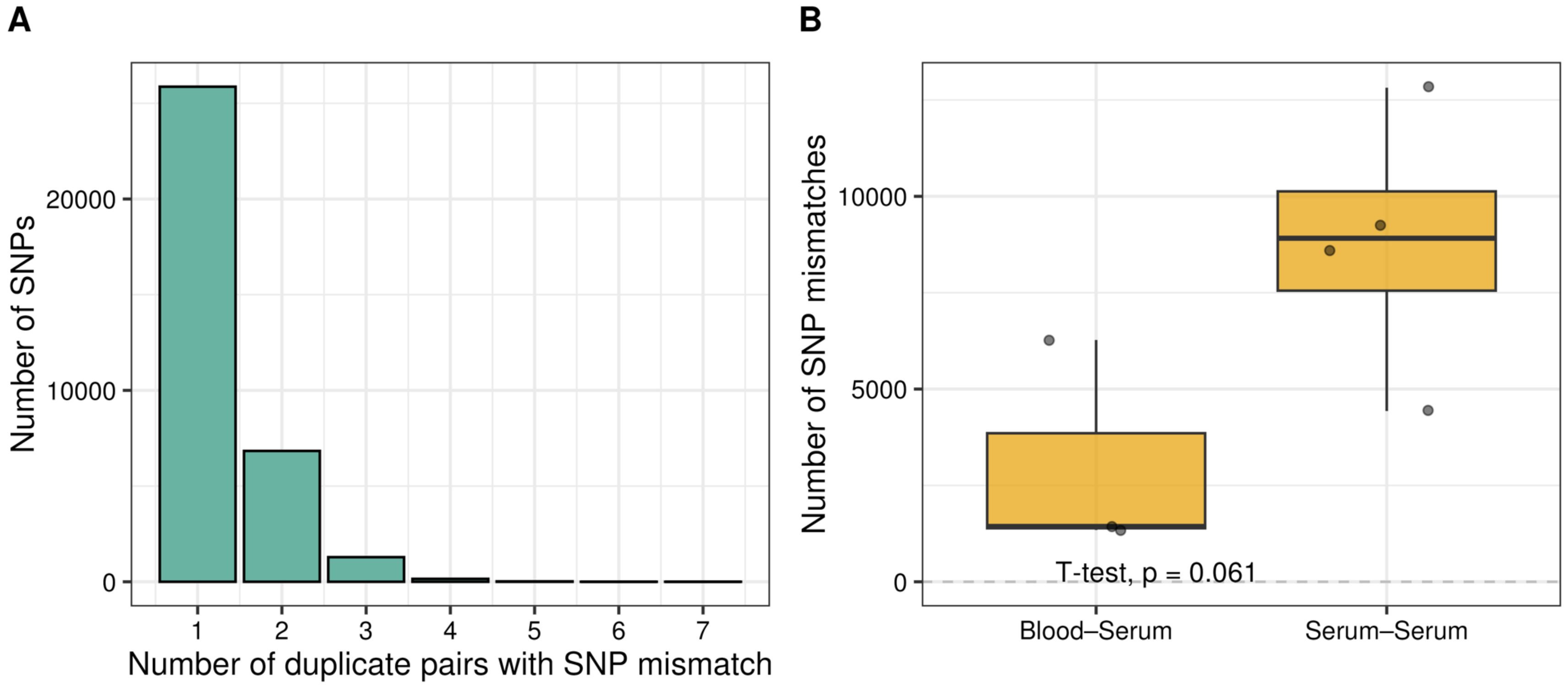
A
Number of SNPs
20000
10000
0
1
2
3
4
5
6
7
Number of duplicate pairs with SNP mismatch
B
Number of SNP mismatches
10000
5000
0
T-test, p = 0.061
Blood–Serum
Serum–Serum

Figure S4

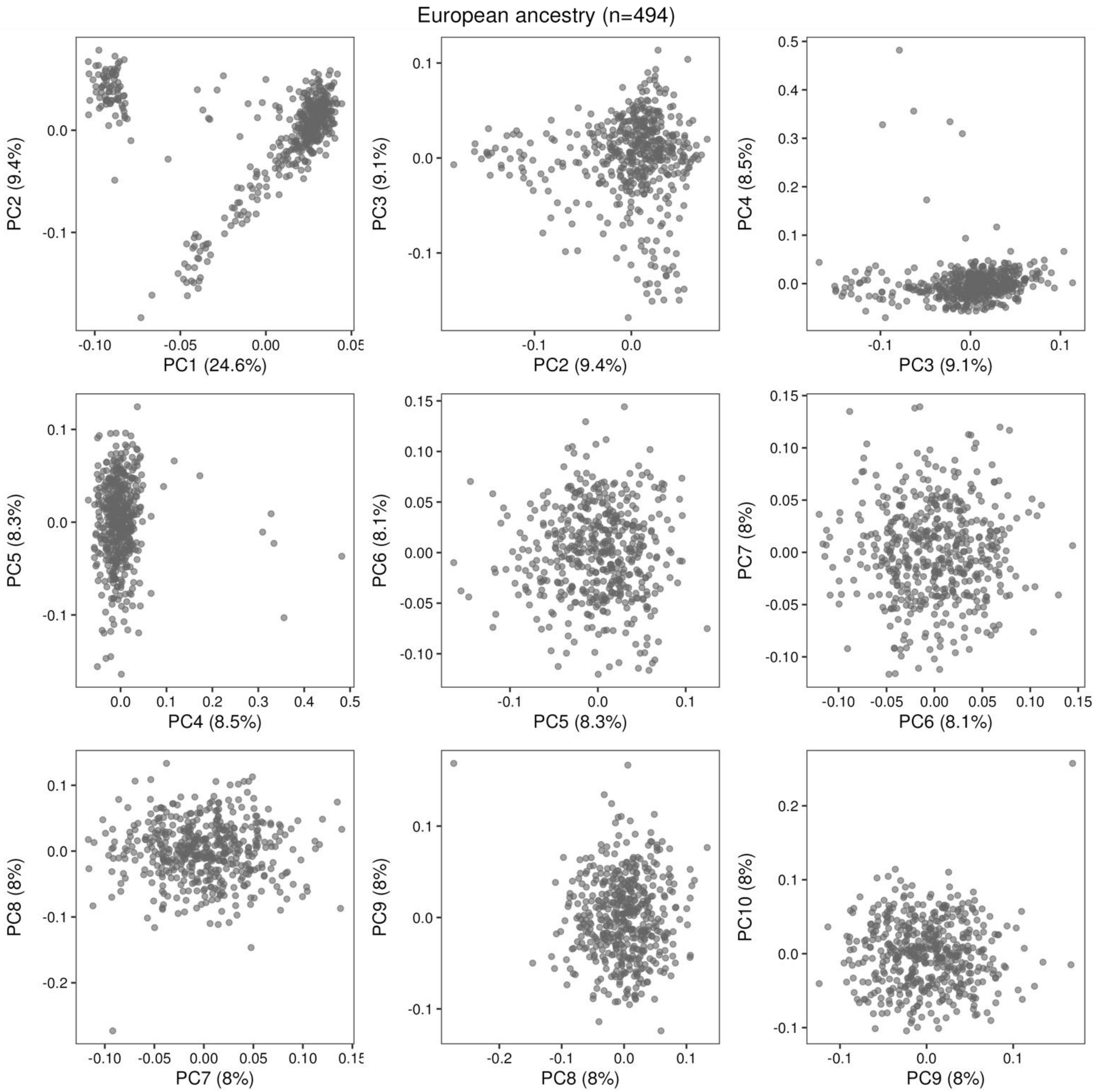
European ancestry (n=494)
PC1 (24.6%)
PC2 (9.4%)
PC3 (9.1%)
PC4 (8.5%)
PC5 (8.3%)
PC6 (8.1%)
PC7 (8%)
PC8 (8%)
PC9 (8%)
PC10 (8%)

Figure S5

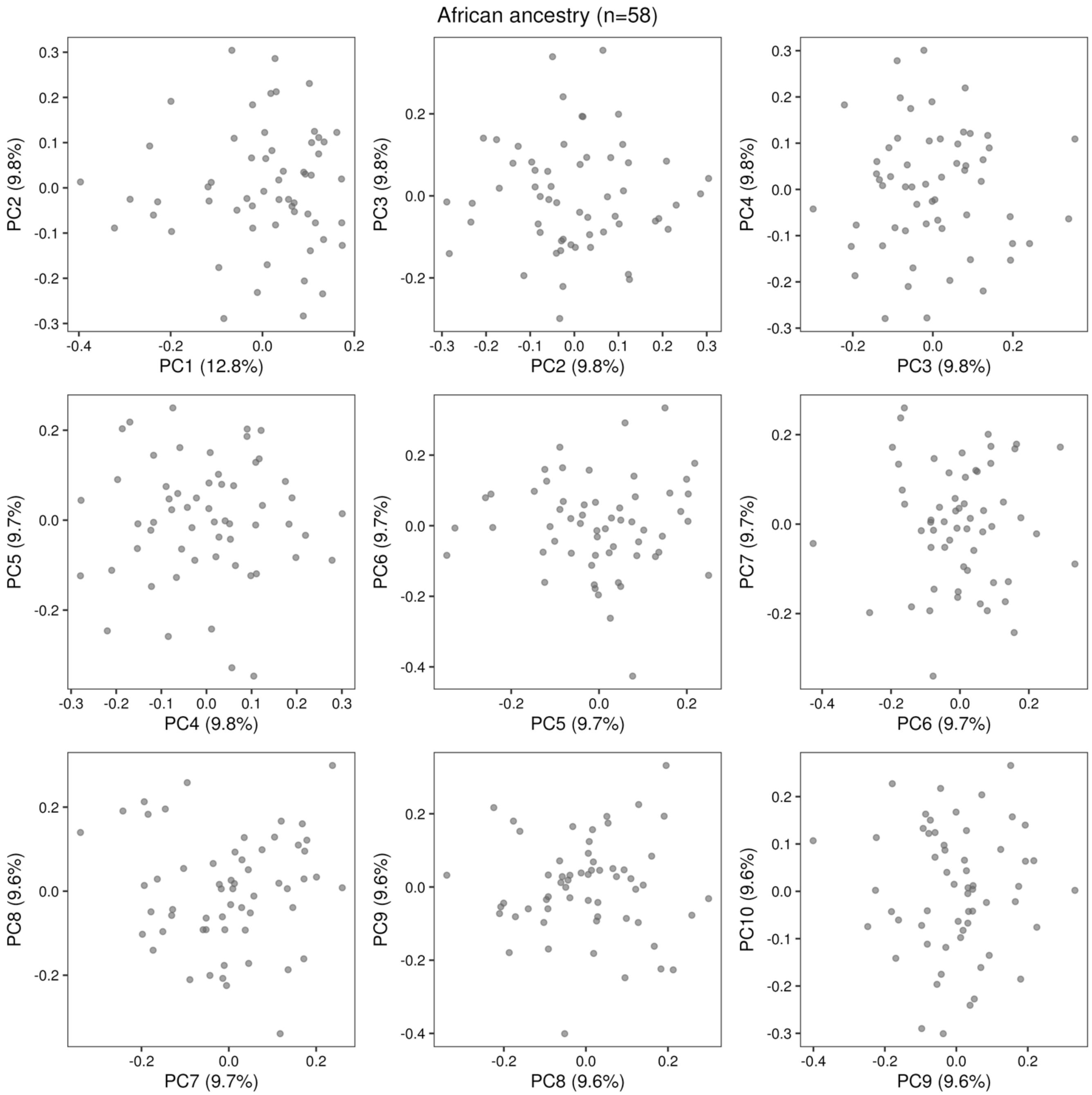
African ancestry (n=58)
PC1 (12.8%)
PC2 (9.8%)
PC3 (9.8%)
PC4 (9.8%)
PC5 (9.7%)
PC6 (9.7%)
PC7 (9.7%)
PC8 (9.6%)
PC9 (9.6%)
PC10 (9.6%)
